# Disrupting the network of co-evolving amino terminal domain residues relieves mitochondrial calcium uptake inhibition by MCUb

**DOI:** 10.1016/j.csbj.2024.12.007

**Published:** 2024-12-12

**Authors:** Danielle M. Colussi, Ryan Grainger, Megan Noble, Taylor Lake, Murray Junop, Peter B. Stathopulos

**Affiliations:** a Department of Physiology and Pharmacology, Schulich School of Medicine and Dentistry, University of Western Ontario, N6A5C1, Canada; b Department of Biochemistry, Schulich School of Medicine and Dentistry, University of Western Ontario, N6A5C1, Canada

**Keywords:** Mitochondrial calcium uniporter (MCU), MCU dominant-negative beta (MCUb), Amino-terminal domain, Divalent cation binding, Feedback regulation, Molecular dynamics, PySCA of residue co-evolution, Solution NMR, X-ray crystallography, Mitochondrial Ca^2+^ uptake

## Abstract

The regulatory mechanisms of the mitochondrial calcium uniporter complex (mtCU), the predominant channel mediating calcium (Ca^2^^+^) flux into the matrix, are critical for bioenergetics and cell fate. The pore-forming components of mtCU are the mitochondrial Ca^2+^ uniporter (MCU) subunit and the MCU dominant-negative beta (MCUb) subunit. Despite both MCU paralogs having conserved Asp-Ile-Met-Glu motifs responsible for Ca^2+^ selectivity, MCUb mediates only low Ca^2+^ conduction and has been characterized as an inhibitory subunit. We previously identified the MCU amino-terminal domain (NTD) as a negative feedback regulator of mtCU upon divalent cation binding but the role of the MCUb-NTD remains unknown. Thus, to gain mechanistic insight into the competing MCU and MCUb functions, we here studied the divalent cation binding properties of the MCU- and MCUb-NTDs that tightly interact within and between tetrameric channels. First, we resolved a high-resolution MCU-NTD crystal structure in the absence of divalent ions at 1.6 Å, using this structure to model the homologous MCUb-NTD. Further, we conducted 1 *μs* all-atom molecular dynamics (MD) simulations in the presence and absence of Ca^2+^ and Mg^2+^ ions, not only finding increased MCU-NTD stability at high temperatures compared to MCUb-NTD but also discrete Ca^2+^-binding sites on the two domains. Remarkably, the distinct Ca^2+^ binding site on the central α-helix of MCUb-NTD was also identified in a functional sector of co-evolving residues, with either direct mutation to the coordinating residues or mutation to a separate site within the sector disrupting Ca^2+^ binding *in silico* and *in vitro* as well as enhancing mitochondrial Ca^2+^ uptake *in cellulo*. Thus, we reveal that matrix Ca^2+^ binding to both the MCU-NTD and MCUb-NTD promote mtCU inhibition through disparate interaction sites, highlighting the evolution of discrete feedback regulation mechanisms to precisely control mtCU function.

## Graphical Abstract

**Figure:**
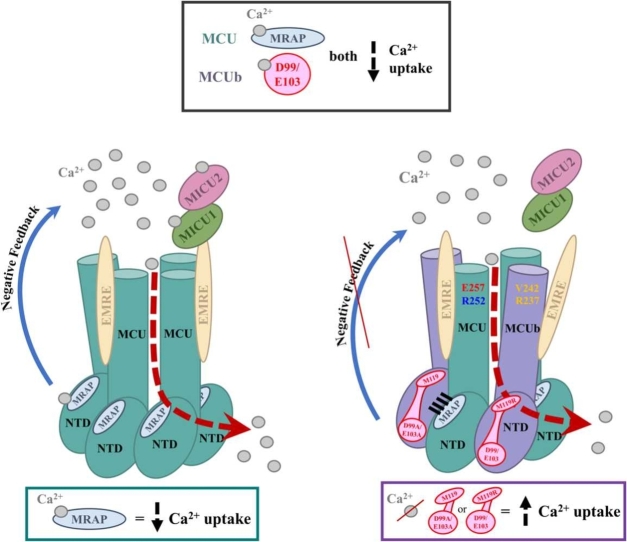

## 1 Introduction

Mitochondrial activities such as ATP production, autophagy and cell death are strongly regulated by matrix calcium (Ca^2+^), and mitochondrial Ca^2+^ uptake influences cytosolic Ca^2+^ fluctuations involved in myriad cell signaling processes [1], [2]. Thus, mitochondrial Ca^2+^ uptake is carefully controlled via entry and efflux mechanisms as well as environment-specific regulation of the involved protein machinery [3], [4], [5], [6], [7], [8]. Upon a physiologic stimulus, microdomains of elevated Ca^2+^ at the outer mitochondrial membrane (OMM) enable Ca^2+^ to flow through nearby voltage-dependent anion channels (VDAC) [2], [9]. Ca^2+^ uptake is then driven into the mitochondrial matrix by the highly negative inner mitochondrial membrane (IMM) potential (*ΔΨ*_*M*_ ≈ −180 mV) established by electron transport chain proton pumping [10], [11]. The mitochondrial Ca^2+^ uniporter complex (mtCU) formed across the IMM is the predominant channel responsible for mediating Ca^2+^ uptake into the matrix [8], [12], [13].

The principal components of mtCU are the pore-forming, mitochondrial Ca^2+^ uniporter (MCU) subunits that oligomerize into tetrameric channels [14], [15]. The conserved Asp-Ile-Met-Glu (DIME) motif in each subunit forms the Ca^2+^ selectivity filter at the intermembrane space (IMS)-exposed channel entrance by symmetric arrangement of the four subunits [16], [17]. Along with MCU, other necessary components of mtCU include mitochondrial Ca^2+^ uptake 1, 2, and 3 (MICU1, MICU2 and MICU3) [18], [19], [20], [21] and essential MCU regulatory (EMRE) proteins involved in channel gating [22]. A paralog of MCU, the MCU dominant-negative beta subunit (MCUb), has been shown to integrate into mtCU channels through amino terminal domain (NTD) interactions [23], exerting a poorly understood inhibitory effect on Ca^2+^ permeation [24], [25]. While previous studies have shown that MCUb does not form a Ca^2+^ permeable channel within planar lipid bilayers [25], the conserved Ca^2+^ selective DIME motif in MCUb has raised questions about the precise inhibitory mechanism of the MCU paralog.

In humans, full-length MCU shares ∼49 % sequence identity and ∼84 % similarity with MCUb, with the matrix-oriented NTDs specifically sharing ∼87 % sequence similarity [26]. Tetrameric mtCU channels self-associate both within and between their NTDs to form larger V-shaped complexes along the curvature of the IMM [13]. However, co-immunoprecipitation assays showed that the MCU-NTD is not essential for MCU oligomerization, and an MCU-NTD deletion (ΔNTD) mutant maintained interactions with both gatekeeping MICU1/2 subunits, analogous to wild-type (WT) [27]. Interestingly, overexpression of MCU_ΔNTD_ in HeLa cells inhibited mitochondrial Ca^2+^ uptake [27], suggesting the NTD may play an autoregulatory role in channel function. Indeed, we and others [28], [29] demonstrated that binding of Ca^2+^ or Mg^2+^ to an MCU regulating acidic patch (MRAP) on the NTD destabilizes domain self-association and inhibits mtCU activity. We also recently showed that the MCU- and MCUb-NTDs interact with ∼nM affinity, with heteromeric assembly favoured over homomeric interactions, resistant to divalent cation perturbation [23]. The importance of this heteromeric NTD interaction has been underscored recently in a study showing the MCUb-NTD is necessary for integration into and inhibition of mtCU [30]. Nevertheless, the lack of full conservation of the three key MCU-NTD MRAP residues (*i.e.* D131, D147, D148) in MCUb-NTD (*i.e.* D116, N132, D133) suggests divergent autoregulatory mechanisms exist involving MCUb-NTD but the mechanisms underlying MCUb regulation remain a major knowledge gap in the field.

Here, we applied X-ray crystallography, homology modeling, molecular dynamics (MD) simulations, *in vitro* biophysical assessments and *in cellulo* mitochondrial Ca^2+^ uptake assays to gain mechanistic insight into the regulatory role of the MCUb-NTD in mtCU activity. We successfully resolved a 1.6 Å atomic-resolution MCU-NTD crystal structure in the absence of divalent cations, which was an ideal starting state to assess how divalent cations affect the structure and dynamics of the NTDs using fs resolution MD simulations. MD trajectories of 1 *μs* revealed increased stability and distinct Ca^2+^ specific coordinating residues for MCU-NTD compared to the homology-modeled MCUb-NTD. One functional protein sector of co-evolving residues was identified using python-based statistical coupling analysis (pySCA) [31], [32] for both the MCU- and MCUb-NTDs, including Ca^2+^ coordinating residues on the central α-helix of MCUb-NTD but excluding MRAP of MCU-NTD. Consistent with pySCA, a mutation (*i.e*. M119R) targeted to the Ca^2+^ regulating sector of MCUb-NTD, on the opposite face of the protein as the Ca^2+^ coordinating residues, resulted in disruption of Ca^2+^ binding at the central α-helix similar to direct mutational variation (*i.e.* D99A/E103A) of the Ca^2+^ coordinating residues, as revealed *in silico* and *in vitro* by circular dichroism (CD), extrinsic 8-anilino-1-napthalene sulfonate (ANS) binding and solution NMR experiments. Excitingly, introducing either M119R or D99A/E103A mutations into full-length MCUb resulted in enhanced histamine-induced mitochondrial Ca^2+^ uptake in HeLa cells. Together, our data highlight that MCU- and MCUb-NTDs are differentially regulated by temperature and divalent cations and reveal that these domains have evolved discrete feedback regulatory input sites that both temper mtCU activity.

## 2 Methods

### 2.1 Construct Generation and Purification

**Fig. 1:**
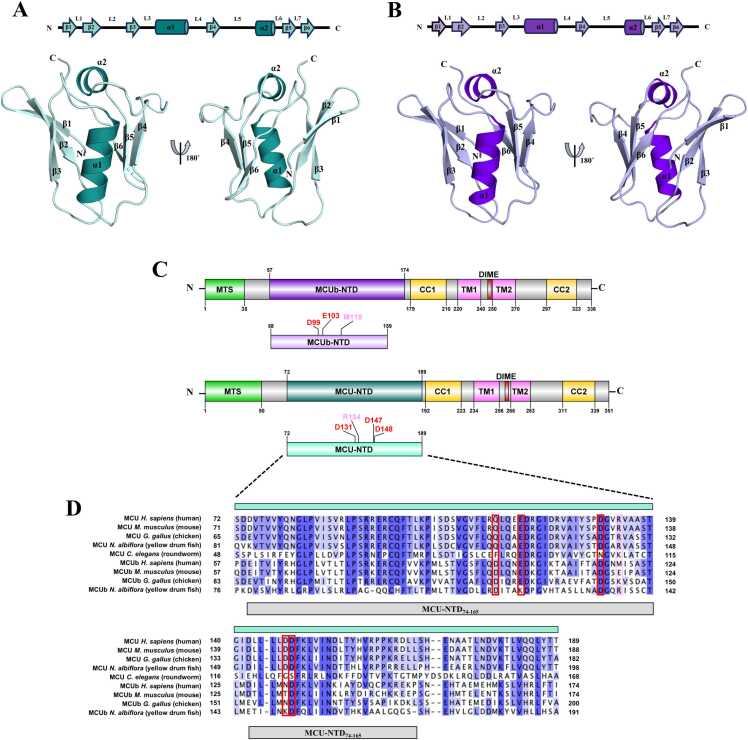
Domain architecture and multiple sequence alignment of human MCU and MCUb proteins. (A) The high-resolution X-ray crystal structure to 1.6 Å resolution of human MCU-NTD (8URG.pdb; teal) in the absence of divalent cations was used as a template to homology model (B) MCUb-NTD (purple). The relative residue regions of the β-grasp-like fold, consisting of two helices, held between two β-sheets for MCU-NTD and MCUb-NTD, respectively, are depicted above and labeled on the three-dimensional structures. Secondary structure was calculated using GROMACS 2022.3 (gmx do_dssp). (C) The domain architecture of human MCUb and MCU are shown with the mitochondrial targeting sequence (MTS, green), amino-terminal domain (NTD, purple for MCUb, teal for MCU), coiled-coil domain −1 and −2 (CC1/2, yellow), transmembrane −1 and −2 (TM1/2, pink), and the Asp-Ile-Met-Glu motif (DIME, red). The residue ranges are shown above or below the respective regions, identified using Uniprot accession Q8NE86 for MCU and Q9NWR8 for MCUb. Below the full-length MCU and MCUb proteins are the respective N-terminal domain (NTD) constructs engineered for this study, highlighting D99, E103 (red) and M119 (light pink) in MCUb-NTD and D131, D147, D148 (red) and R134 (light pink) in MCU-NTD. (D) Multiple sequence alignment of MCU and MCUb paralogs from higher to lower order organisms. Sequences for human MCU (NCBI, NP_612366.1) and MCUb (NCBI, NP_060388.2), *Mus musculus* MCU (NCBI, NP_001028431.2) and MCUb (NCBI, NP_080055.3), *Gallus gallus* MCU (NCBI, XP_015143713.1) and MCUb (NCBI, XP_046772790.1), *Nibea albiflora* MCU (NCBI, KAG800000018.1) and MCUb (NCBI, KAG8005890.1), and *Caenorhabditis elegans* MCU (NCBI, NP_500892.1) were aligned using Clustal Omega with defaults [48] and annotated in Jalview [73]. Boxes highlight the location of D99, E103 (red) based on human MCUb, and D131, D147, D148 (red) based on human MCU, with M119 in human MCUb corresponding to R134 in human MCU (light pink). The light teal colored box above the human MCU sequence marks the boundary of the recombinant human MCU-NTD construct engineered for this study, and the grey box below the MCUb *N. albiflora* sequence corresponds to the resolved residues of our new MCU-NTD crystal structure (8URG.pdb). The residue numbers are shown at the left and right of each entry line, with darker blue shading corresponding to increased conservation of positions.

A stably folded MCU-NTD construct encompassing residues 72–189 (*i.e.* MCU-NTD_WT_) was generated based on a multiple sequence alignment of MCU proteins from higher to lower order organisms (roundworm, fruit fly, fish, chicken, human, and mouse sequences) and cloned into a pET-28a vector [29]. The MCUb-NTD residues 58–159 (*i.e.* MCUb-NTD_WT_) construct cloned into pET-28a was then designed to encompass the corresponding MCU-NTD_WT_ residues, with the 14 unstructured residues at the C-terminus omitted (Fig. 1A). The D99A/E103A mutant (*i.e.* MCUb-NTD_D99A/E103A_) and M119R mutant (*i.e.* MCUb-NTD_M119R_) were separately introduced into pET-28a MCUb-NTD_WT_ by PCR-site directed mutagenesis. The complementary mutagenic primers were 5’-GTTCATTCCTTCAGGCCCTACAAAATGCAGATAAGGGTATC-3’ and 3’-CAAGTAAGGAAGTCCGGGATGTTTTACGTCTATTCCCATAG-5’ for MCUb-NTD_D99A/E103A_, and 5’-ACAGCAGATGGCAACCGGATTTCAGCTTCTACC-3’ and 3’-TGTCGTCTACCGTTGGCCTAAAGTCGAAGATGG-5’ for MCUb-NTD_M119R_.

The MCU-NTD and MCUb-NTD wild-type (WT) and mutant proteins were each transformed in BL21 (DE3) codon plus *Escherichia coli* cells and grown in Luria-Bertani (LB) broth supplemented with kanamycin (60 µg mL^−1^) at 37 ˚C until optical density at 600 nm reached ∼0.6–0.8, when 350 µM of isopropyl β-D-1-thiogalactopyranoside (IPTG) was added to induce expression for 17 hr at 24.5 ˚C. Harvested cells were purified under denaturing conditions with nickel-nitrilotriacetic acid (Ni^2+^-NTA) affinity chromatography. The proteins were refolded in 20 mM Tris (pH 8.5), 200 mM NaCl, and 2 mM DTT by dialysis with a 6–8 kD MWCO membrane (Thermo Fisher Scientific), before cleaving the N-terminal His_6_ tags with ∼1 unit of bovine thrombin (Sigma-Aldrich) per mg of protein. Finally, the proteins were purified by size exclusion chromatography (SEC) through a Superdex 200 10/300 GL column connected to an AKTA pure FPLC system (GE Healthcare) in 20 mM Tris (pH 8.5), 150 mM NaCl, and 1 mM DTT for use in X-ray crystallography, extrinsic ANS fluorescence, and far-UV CD experiments. The MCUb-NTD_WT_ and mutant constructs were also uniformly ^15^N-labelled and expressed in M9 minimal media for NMR spectroscopy experiments. The purification procedure remained the same as the unlabeled protein, except the SEC running buffer was 20 mM HEPES, 150 mM KCl, and 1 mM DTT (pH 8.0 for MCUb-NTD_WT_ and MCUb-NTD_D99A/E103A_, and pH 8.5 for MCUb-NTD_M119R_).

For our GCaMP6f mitochondrial Ca^2+^ uptake experiments, we utilized the pCMV-MCU-eGFP plasmid from our previous work [29]. The mCherry was cloned out of pCMV-mCherry-STIM1 [33] into a unique C-terminal *Eco*RI site and introduced into pcDNA-MCUb (Genescript) by PCR site-directed mutagenesis to generate the pcDNA-MCUb-mCherry plasmid. The cDNA sequence encoding residues 79–160 was excised by digestion and re-ligated using an introduced Xbal site at A159 and an endogenous *Xba*I site at S77/R78 to generate the pcDNA-MCUb_ΔNTD_-mCherry construct. The MCU, MCUb and MCUb_ΔNTD_ constructs were then cloned out of the pcDNA vectors into a pEGFP-N1 vector using *Hin*dIII and *Kpn*I sites. The C-terminal EGFP of these vectors were replaced with GCaMP6f from the pCMV-mito-4x-GCaMP6f vector [34] using *Kpn*I and *Mfe*I to generate the pEGFP-MCU-GCaMP6f, pEGFP-MCUb-GCaMP6f, and pEGFP-MCUb_ΔNTD_-GCaMP6f constructs [23]. The pEGFP-MCUb_D99A/E103A_-GCaMP6f and pEGFP-MCUb_M119R_-GCaMP6f constructs were generated by PCR site-directed mutagenesis with pEGFP-MCUb-GCaMP6f using the complementary mutagenic primers indicated above.

### 2.2 MCU-NTD_74–165_ crystallization

MCU-NTD_WT_ and MCUb-NTD_WT_ constructs were purified in 20 mM Tris (pH 8.5), 150 mM NaCl, 1 mM DTT and concentrated to ∼200 µM using an Amicon Ultra-15 10,000 molecular weight cut-off centrifugal concentrator. Subsequently, a 200 µM final concentration of both samples were combined to produce a 600 µL equimolar mixture. The samples equilibrated at 4 ˚C for 72 hr prior to crystal tray set-up. The three-dimensional structure of the MCU-NTD_WT_ was determined by X-ray crystallography using hanging drop vapour diffusion in a 24-well crystallization plate (NeXtal). The precipitant consisted of 50 mM imidazole (pH 8.0), 150 mM Li_2_SO_4_, 30 % (w/v) polyethylene glycol (PEG) 3000, and 4 % (v/v) formamide at a 1 μL to 2 μL drop ratio of protein to precipitant, over the reservoir solution of 1.5 M (NH_4_)_2_SO_4_. The crystal grew at 20 ˚C, before being flash-frozen in the cryo-jet stream held at 100 K.

### 2.3 X-ray diffraction and structure refinement

The diffraction data of the MCU-NTD_WT_ crystal was collected at 100 K using synchrotron X-ray sources on CLSI beamline 08B1–1 with a DECTRIS PILATUS3 6 M diffraction detector at the Canadian Light Source (CLS, Canada). Diffraction data was collected at a resolution of 1.6 Å using a single-wavelength of 1.5212 Å. The diffraction data was then processed with Phenix by molecular replacement using the previously resolved MCU-NTD_WT_ structure in the presence of Mg^2+^ (5KUJ.pdb; [29]) as a template. The data collection and refinement statistics are provided in Table 1.

**Table 1:** Crystallization data and refinement statistics for MCU-NTD_74–165_.

	**MCU-NTD**_**74–165**_
	**PDB ID: 8URG**
**Data collection**	
Space group	P6_5_
Wavelength (Å)	1.5212
X-ray source^a^ and detector	CLSI 08B1–1, DECTRIS PILATUS3 6 M
Unit cell: *a, b, c* (Å)	55.52, 55.52, 69.11
α, β, γ (°)	90, 90, 120
Resolution range (Å)^b^	28.06–1.6 (1.657–1.6)
Unique reflections	14191 (925)
Completeness (%)	88.74 (58.21)
Mean (*I/σI*)	13.3 (1.0)
Wilson B-factor	16
*R*_merge_ (%)	7.8 (188.5)
*R*_meas_ (%)	8.7 (217.5)
*R*_pim_ (%)	3.9 (105.9)
CC1/2	0.998 (0.470)
**Model and refinement**	
Reflections used in refinement	14191 (925)
Reflections used for *R*_free_	723 (55)
*R*_work_	0.1988 (0.4619)
*R*_free_	0.2269 (0.4475)
Number of non-hydrogen atoms	854
Macromolecules	728
Solvent	126 water
Protein residues	92
RMSD bond lengths (Å)	0.010
RMSD bond angles (°)	1.33
Ramachandran favoured (%)	98.89
Ramachandran allowed (%)	1.11
Ramachandran outliers (%)	0.00
Rotamer outliers (%)	1.20
Clashscore	2.05
Average B-factor	22.10
Macromolecules	20.52
Solvent	31.27
Number of TLS groups	1

a Beamline 08B1–1 at the Canadian Light Source Inc. (CLSI) in Saskatchewan, Canada.

b Values in parentheses are for the highest-resolution shell.

### 2.4 Homology modeled MCUb-NTD_59–150_ structure

The primary amino acid sequence of the human MCUb protein was retrieved from the NCBI protein database (NP_060388.2). Homology modeling was then carried out for the MCUb-NTD_WT_ against the MCU-NTD_WT_ template crystal structure (8URG.pdb) using Modeller [35]. The MCU-NTD_WT_ template crystal structure consisted of residues 74–165, corresponding to residues 59–150 in MCUb. The final model of the MCUb-NTD_WT_ was generated by creating 10 models and selecting the final model with the lowest DOPE (discrete optimized protein energy) score [36].

### 2.5 MD simulations

Molecular dynamics (MD) simulations of MCU-NTD_WT_ and MCUb-NTD_WT_ and mutant monomer structures were conducted using GROMACS 2022.3 package [37] and the Digital Alliance of Canada supercomputing resources. MCU-NTD and MCUb-NTD mutant monomer structures were generated using the mutagenesis plugin in PyMOL 2.5.2 (Schrödinger LLC**)**. Molecular topologies were generated based on the solvated lysozyme tutorial [38] using the OPLS-AA/L all-atom force field [39] that includes parameters for both Ca^2+^ and Mg^2+^ [40], and neutral N- and C-termini. The simple point charge SPC216 water model was used to solvate the systems in a cubic box with a solute-box distance of 1.0 nm. One Ca^2+^ or Mg^2+^ ion was added into the system to replace a water molecule, resulting in 8 mM divalent cations within the solvated system, with 5 Ca^2+^ or Mg^2+^ ions added into the system to saturate binding sites for 40 mM divalent cations. Water was replaced with Cl^-^ ions to neutralize net charges. The solvated, electroneutral systems were then relaxed through steepest descent energy minimization. Systems were equilibrated for the NVT ensemble (*i.e.* fixed number of atoms, volume, and temperature) at a constant temperature of either 310 K or 320 K (*i.e.* ∼37 ˚C or ∼47 ˚C, respectively) for 100 ps using the modified Berendsen’s thermostat algorithm [41]. The second equilibration phase was under NPT ensemble (*i.e.* fixed number of atoms, pressure, and temperature) for 100 ps using the Parrinello-Rahman barostat pressure [42] of 1 bar. The final MD simulations were conducted for 1 *μs* under NPT ensemble conditions.

### 2.6 Analysis of MD trajectories

Analyses of the MD simulation trajectories were preformed using the analysis tools in the GROMACS 2022.3 package. Root-mean-square deviation (RMSD) were calculated for the Cα atoms of the total protein (gmx rms) and comparing the central α-helix region (gmx helix) to an ideal α-helix [43], only processing every 10th frame. The rotation and translation of proteins was removed from the final trajectory files to calculate S^2^ order parameters. An index file was created to define N-H bonds for each residue, excluding the N-terminal and Pro residues, to calculate autocorrelation using 2nd order Legendre polynomial (gmx rotacf) before calculating the S^2^ order parameters using a perl script. The distances between the oxygen atoms of the conserved divalent cation coordinating residues of MRAP in MCU-NTD and MCUb-NTD (*i.e*. D131 and D148 in MCU correspond to D116 and D133 in MCUb) and the coordinating residues of the central α-helix in MCUb-NTD (*i.e*. D99 and E103) were calculated over the simulation (gmx distance). The number of interactions over the simulations between each divalent cation in the respective system and specified residues of interest was calculated (gmx mindist).

To visualize the 1 *μs* trajectory files as movies, periodic boundary conditions were first set to put atoms that jump across the box back in (-pbc nojump) and then center the mass of molecules in the box (-pbc mol). The rotation and translation of the proteins were removed on a frame-to-frame basis (-fit rot+trans), processing every 10th frame. The mp4 file movie outputs were then generated in ChimeraX [44]. Snapshots of the proteins over each simulation were visualized using PyMoL 2.5.2 (Schrödinger LLC**)**. The water molecules were removed for visualization. Notably, Mg^2+^ has been shown to bind tightly to 6 water molecules, with the coordination number of Ca^2+^ shown to vary experimentally from 6 to 10 [45]. Thus, Ca^2+^ is able to shed its solvation shell more readily than Mg^2+^ due to a higher solvation free energy of Mg^2+^ in water [45].

### 2.7 Statistical coupling analysis

The python-based statistical coupling analysis (pySCA) began with assembling a large and diverse multiple sequence alignment for the MCU and MCUb protein family [46] using BLASTp [47]. The sequence alignment with MCU used as the query sequence resulted in 1948 sequences and 91 positions, with 167 total effective sequences after filtering out 90–100 % sequence identity to human MCU. When MCUb was used as the query sequence, 2226 sequences and 91 positions resulted, with 168 effective sequences after sequences with 90–100 % identity to human MCUb were removed. The complete set of sequences was aligned using Clustal Omega [48] and further truncated to match the residues from MCU-NTD_74–165_ refined crystal structure. Pre-processing of the alignment was conducted with the scaProcessMSA script, and sector definition utilized the scaCoreMSA and scaSectorID python scripts. The pySCA sector analysis was conducted in a jupyter notebook using script adapted from the pySCA tutorials [31].

### 2.8 Extrinsic ANS fluorescence

Extrinsic 8-anilino-1-napthalene sulfonate (ANS) (Sigma) fluorescence spectra were acquired using a Cary Eclipse spectrofluorometer (Agilent/Varian) at 25 ˚C with 0.25 mg mL^−1^ of protein in 20 mM Tris (pH 8.5), 150 mM NaCl, 1 mM DTT, and 50 mM ANS using a 600 *μ*L quartz cuvette. The excitation wavelength used was 372 nm, with excitation and emission slits of 10 and 20 nm, respectively. Extrinsic ANS fluorescence emission spectra were acquired between 400 and 600 nm to monitor divalent cation-induced changes in solvent-exposed hydrophobicity of each protein, with two overlaid spectra averaged for both Ca^2+^-free and 5 mM CaCl_2_-supplemented samples directly into the cuvette, with a 5 min equilibration. The photomultiplier tube detector was set to 600 V for MCUb-NTD_WT_ and 500 V for both MCUb-NTD_D99A/E103A_ and MCUb-NTD_M119R_. The voltage difference was normalized by acquiring similar spectra in the absence of protein, with negligible effects of CaCl_2_ found on free ANS fluorescence.

### 2.9 Far-UV CD spectroscopy

Far-UV CD spectra were acquired using a Jasco J-810 CD spectrometer with an electronic Peltier temperature controller (Jasco). Each spectrum was an average of 3 scans acquired between 200 and 240 nm at 20 ˚C using a 1 mm path length quartz cuvette with a speed of 20 nm min^−1^, data pitch of 1 nm, and response time of 8 sec. After acquiring divalent cation-free spectra at 0.35 mg mL^−1^, 5 mM CaCl_2_ was added to the same samples, and spectra were re-acquired after a 5 min equilibration.

### 2.10 Intrinsic fluorescence binding curves

Intrinsic fluorescence experiments were performed using a Cary Eclipse spectrofluorometer (Agilent/Varian) at 22.5 ˚C with an excitation wavelength of 280 nm and excitation and emission slit widths of 5 and 10 nm, respectively. A total of 8 emission spectra were acquired from 295 to 400 nm on 0.25 mg mL^−1^ MCUb-NTD_WT_ and MCU-NTD_WT_ samples in 20 mM Tris (pH 8.5), 150 mM NaCl and 1 mM DTT, titrating in CaCl_2_ from 0 to 5 mM. Spectra were acquired using a 3 mm path-length quartz cuvette. Control experiments without CaCl_2_ were conducted for both MCUb-NTD_WT_ and MCU-NTD_WT_ and subtracted from the binding curves to account for protein adsorption. Binding curves were then fit to a one-site binding model in R (version 4.2.1), taking into account protein concentration, to extract the apparent equilibrium dissociation constant for Ca^2+^ (K_d, Ca2+_).

### 2.11 Solution NMR spectroscopy

Solution NMR spectroscopy experiments were performed using a 600 MHz Bruker 600 spectrometer (Varian). ^1^H-^15^N-heteronuclear single quantum coherence (HSQC) spectra for MCUb-NTD_WT_ and mutant proteins were acquired at 20 ˚C using 1024 points in the ^1^H dimension with a 8000 Hz ^1^H sweep width, and 32 transients and 64 increments in the ^15^N dimension with a 1800 Hz ^15^N sweep width. Each sample was acquired in the absence of divalent cations before supplementation with 40 mM CaCl_2_. All samples were in 20 mM HEPES, 150 mM KCl, 1 mM DTT (pH 8.0 for MCUb-NTD_WT_ and MCUb-NTD_D99A/E103A_, pH 8.5 for MCUb-NTD_M119R_) in the presence of 50 μL of DSS in D_2_O and 10 μL of 100 % D_2_O. The NMR data was processed using NMRPipe [49] and ^1^H-^15^N peak chemical shift perturbations (CSPs) and intensity changes, comparing each sample in the absence and presence of 40 mM CaCl_2_, were done with XEASY [50].

### 2.12 Mitochondrial Ca^2+^ uptake in permeabilized HEK293T cell experiments

HEK293T cells were grown on 10 cm dishes in DMEM with high glucose (Wisent) supplemented with 10 % (v/v) FBS (Sigma-Aldrich) and 1 % (v/v) penicillin/streptomycin (Wisent) at 37 °C with 5 % CO_2_/95 % air. The cells were cultured into 35 mm dishes for transfection at ∼70 % confluency using PolyJet In Vitro DNA Transfection Reagent (SignaGen) according to manufacturer guidelines. The cells were transiently transfected with 1 µg of either pEGFP-MCU-GCaMP6f, -MCUb-GCaMP6f, or -MCUb_ΔNTD_-GCaMP6f fusion constructs.

Only once the dish reached ∼90 % confluency (typically after ∼24 h) to ensure similar numbers of cells in each assay, the cell medium was aspirated, and the cells were washed once with 1 mL intracellular buffer (IB) [20 mM HEPES (pH 7), 10 mM NaCl, 130 mM KCl, 2 mM K_2_HPO_4_, 5 mM succinate, 5 mM malate, and 1 mM pyruvate]. The cells were lifted from the dish using 2 mL of IB, and the cell mixture was placed in a 2 mL plastic cuvette with a small stir bar. GCaMP6f fluorescence was observed using a PTI QuantMaster spectrofluorometer (Horiba) equipped with electronic temperature control set to 22.5 °C, with an excitation wavelength of 475 nm and emission wavelength of 515 nm. The excitation and emission slit widths were 7.5 nm and 10 nm, respectively. The cells were incubated for five minutes before measurement with the addition of 2.5 μM digitonin and 2.5 mM EGTA. Fluorescence intensity was then measured for 190 s with 2.5 mM CaCl_2_ added at 90 s. The data was smoothed in Excel with the exponential smoothing analysis tool, with a damping factor of 0.93. The relative change in GCaMP6f fluorescence was then calculated by F/F_0_, with F and F_0_ representing the fluorescence at a given time point and mean baseline fluorescence from 70 to 85 s, respectively. The maximal change in GCaMP6f fluorescence was calculated by F/F_0_, with F representing the mean final fluorescence from 165 to 185 s and F_0_ representing the mean baseline fluorescence from 70 to 85 s, respectively. Ratios over 1.2 were removed from the analysis, along with a Ru360 insensitive jump present between 90 and 92 s observed in the pEGFP-MCU-GCaMP6f experiments. Welsch’s ANOVA and Games-Howell’s post-hoc test were used due to unequal variances between groups.

### 2.13 Mitochondrial Ca^2+^ uptake in intact HeLa cell experiments

The HeLa cells were cultured as previously described on 35 mm No. 1S glass bottom dishes (Matsunami). The cells were transiently transfected with 1 µg of either pEGFP-MCUb-GCaMP6f, -MCUb_D99A/E103A_-GCaMP6f, or -MCUb_M119R_-GCaMP6f fusion constructs. Once the cells reached ∼90 % confluency after 24 hr, the cell medium was aspirated, and the cells were washed with 2 mL of Ca^2+^-free HEPES buffered saline solution (HBSS). GCaMP6f fluorescence was assessed using a Nikon Diaphot microscope equipped with a 40x objective (Nikon Fluor 40/1.30 Oil Ph4) in intact HeLa cells, focused on a cluster of ∼2–5 cells. Fluorescent excitation and emission intensity detection was driven through a Felix Gx software (version 4.9)-controlled PTI RatioMaster RM50 system equipped with a D104 photometer (Horiba). The excitation slit widths were set to 7.5 nm, and an excitation wavelength of 470 nm was used, with the excitation and emission light passed through a AT/EGFP/FCY2/AF488 filter set (Chroma). Once the dish was mounted, the cells were perfused with 3 mL of Ca^2+^-free HBSS and equilibrated for 10 min before mitochondrial Ca^2+^ uptake measurements.

After a 60-second baseline measurement, 3 mL of 2.0 mM CaCl_2_ and 2.5 μM histamine in HBSS was perfused through the dish, and GCaMP6f fluorescence was monitored for an additional 4 min with a 0.2 s averaging time and 5 points per s. Following each experiment, the cells were perfused with 10 mL of Ca^2+^-free HBSS with a 10 min incubation period before measurement on a separate ∼2–5 cells. A maximum of three histamine responses per dish were acquired per day over a maximum of two days. Raw data was smoothed in Excel with the exponential smoothing analysis tool, with a damping factor of 0.93. Data was normalized using F/F_0_, indicating the relative change in GCaMP6f fluorescence, with F and F_0_ representing the fluorescence at a given time point and mean baseline fluorescence from 52 to 60 s, respectively. The maximal change in GCaMP6f fluorescence was calculated by F/F_0 max_ – F/F_0 min_.

### 2.14 Epifluorescence microscopy

Epifluorescence images of live HeLa cells expressing MCUb-GCaMP6f, MCUb_ΔNTD_-GCaMP6f, MCUb_D99A/E103A_-GCaMP6f and MCUb_M119R_-GCaMP6f were acquired using a Zeiss AXIO Observer D1 Inverted Microscope equipped with a Sutter Lambda 721 LED Optical Beam Combining System and Zeiss Axiocam 807 mono CCD camera. Images were captured through a Zeiss 63 × /1.4 Plan Apochromat oil immersion objective using the OBC-480 LED for GCaMP6f and OBC-560 LED for MitoTracker Red (Invitrogen) excitation. Cells were cultured on 35 mm No1S glass bottom dishes (Matsunami) and transfected exactly as specified for the HEK293T cells above. Prior to imaging at ∼90 % confluence, cells were incubated with 250 nM MitoTracker Red CMXRos (Invitrogen) for 45 min, washed with 2 × 1 mL of PBS and bathed in 5 mM CaCl_2_ containing HBSS.

### 2.15 Statistical analysis

Statistical analysis consisted of a two-sample F-test for variances of the distances measured between divalent cation coordinating residues. Welsch’s ANOVA and the Games-Howell post-hoc test were used when comparing more than two sample groups with unequal variances. One-way ANOVA and Tukey’s HSD post-hoc test were used when comparing more than two sample groups with equal variances. A paired *t*-test was used when comparing the same sample before and after supplementation with CaCl_2_. Statistical analyses were conducted in GraphPad Prism (4.02) or R (version 4.2.1).

## 3 Results

### 3.1 The MCU-NTD MRAP is not conserved in MCUb-NTD

Previously, we determined several high-resolution X-ray crystal structures of WT MCU-NTD in the presence and absence of divalent cations [29]. Here, we re-crystallized the human MCU-NTD in the absence of divalent cations, determining a structure to 1.6 Å resolution (Fig. 1A); moreover, this structure is the highest-resolution WT MCU-NTD structure we have achieved to date in the absence of divalent cations, providing the best starting point to investigate the effects of Ca^2+^ and Mg^2+^ binding on conformation and dynamics using *in silico* simulations. Given the high sequence similarity to MCUb-NTD and since no high-resolution structure exists for MCUb, this new structure is also the best template to homology model MCUb-NTD (Fig. 1B-1D). Similar to our previous crystal structures, the present WT MCU-NTD shows a β-grasp-like fold, consisting of two helices, held between two β-sheets (Fig. 1A). The C-terminus (*i.e.* residues 166–189) is not resolved, suggesting the region is dynamic under our crystallization conditions. The central α1-helix (residues 108–118) is approximately perpendicular to the second capping α2-helix (residues 141–146). The antiparallel β1, β2 and β3 strands of the first β-sheet are made up of residues 76–80, 83–88 and 97–100, respectively, while the antiparallel β4, β5 and β6 strands consist of residues 125–128, 149–153 and 156–160, respectively. Our Modeller-determined homology model defines these conserved motifs as follows for MCUb-NTD: α1, residues 93–103; α2, residues 126–131; β1, residues 61–65; β2, residues 68–74; β3, residues 81–85; β4, residues 110–114; β5, residues 134–138; β6 residues 141–145 (Fig. 1B). Notably, the residues that make up MRAP in human MCU-NTD (*i.e.* D131, D147, D148) are not fully conserved in human MCUb-NTD at the primary (*i.e.* D116, N132, D133) and three-dimensional (3D) structural levels (Figs. 1C and 1D).

### 3.2 High temperature stabilizes MCU-NTD but destabilizes MCUb-NTD

**Fig. 2:**
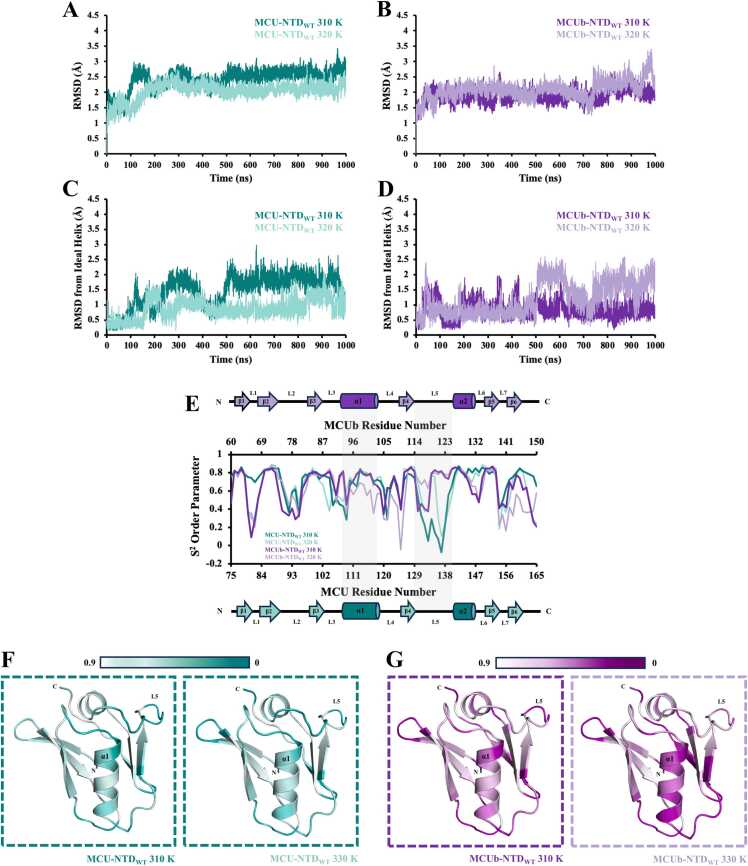
Temperature-dependent effects on conformational change and flexibility of the MCU- and MCUb-NTDs. The average root-mean-square deviation (RMSD) over the 1 *μs* simulations of the 92 Cα atoms for (A) MCU-NTD_WT_ and (B) MCUb-NTD_WT_ was calculated at 310 K (∼37 °C) and 320 K (∼47 °C). RMSD of the central helix region was then determined relative to an ideal helix for the Cα atoms for (C) MCU-NTD_WT_ and (D) MCUb-NTD_WT_ at 310 K (∼37 °C) and 320 K (∼47 °C). (E) Order parameters (S^2^) were calculated as a measure of motion restriction for the backbone N-H bonds of MCU-NTD_WT_ and MCUb-NTD_WT_ at 310 K (∼37 °C) and 320 K (∼47 °C). Schematic diagrams indicating the localization of the secondary structure in MCUb-NTD_WT_ and MCU-NTD_WT_ are shown above and below the S^2^ order parameter plot, respectively, with gray boxes indicating regions with notable differences in S^2^ between MCU and MCUb. Gradients of the S^2^ order parameters for (F) MCU-NTD_WT_ and (G) MCUb-NTD_WT_ were plotted on the three-dimension structures using PyMOL 2.5.2 (Schrödinger LLC), from white to dark teal (MCU) or purple (MCUb) to highlight least to most dynamic residues. In (*A-G*), MCU-NTD_WT_ at 310 K (∼37 °C) and 320 K (∼47 °C) is shown in teal and light teal, respectively, and MCUb-NTD_WT_ at 310 K and 320 K is shown in purple and light purple, respectively.

During periods of high respiratory chain activity and ATP production, it has been suggested that human mitochondria can heat up to ∼50 ˚C [51], [52]. Thus, we first evaluated how temperature affected the conformational dynamics of our MCU- and MCUb-NTD structures using all-atom MD simulations. MD simulations were performed in GROMACS using the OPLS-AA/L force field with fs temporal resolution over 1 *μs* total simulation times. Analysis of the Cα backbone atom RMSDs at 310 K (∼37 °C) and 320 K (∼47 °C) was used to compare temperature-dependent structural stability and conformational changes, with reduced RMSD over the course of a simulation taken as increased structural stability [53], [54]. The average RMSD for the 92 Cα atoms over the 1 *μs* simulation conducted at 310 K (∼37 °C) was higher for MCU-NTD compared to MCUb-NTD (*i.e.* 2.4 versus 1.9 Å, respectively) (Figs. 2A and 2B; Table 2, Table 3). Unexpectedly, when the temperature was increased to 320 K (∼47 °C), the MCU-NTD average RMSD decreased by ∼0.4 Å, in contrast to the MCUb-NTD RMSD that increased by ∼0.2 Å over the simulation time (Figs. 2A and 2B; Table 2, Table 3). The higher temperature promoted a more ideal central helix geometry for MCU-NTD (*i.e.* decreased RMSD by ∼0.6 Å relative to an ideal helix) while the central helix of MCUb-NTD deviated more from ideality at the higher temperature (*i.e.* increased RMSD by ∼0.4 Å) (Figs. 2C and 2D; Table 2, Table 3**;** Videos S1-S4).

**Table 2:** Extracted MD simulation parameters for all MCU-NTD systems obtained after 1 *μs*.

	**Temperature**		**Divalent Cation Concentration**				**MRAP Mutation**	**Sector Mutation**	
	**MCU** **310 K**	**MCU** **320 K**^c^	**MCU** **+ 1 Ca**^**2+**^	**MCU** **+ 5 Ca**^**2+**^	**MCU** **+ 1 Mg**^**2+**^	**MCU** **+ 5 Mg**^**2+**^	**MCU** **D131A/D147A/D148A** **+ 1 Ca**^**2+**^	**MCU R134M**	**MCU R134M** **+ 1 Ca**^**2+**^
**RMSD –** **Total protein (Å)**^a^	2.4 ± 0.3	2.0 ± 0.3	2.2 ± 0.4	3.1 ± 0.8	2.1 ± 0.2	2.1 ± 0.2	3.2 ± 0.4	1.6 ± 0.1	2.4 ± 0.4
**RMSD –** **α-helix 1 (Å)**^a^	1.5 ± 0.5	0.9 ± 0.3	1.0 ± 0.3	2.3 ± 1.0	0.6 ± 0.1	0.6 ± 0.2	0.5 ± 0.1	0.8 ± 0.2	1.4 ± 0.4
**Residue-based S**^**2**^**order parameter**	0.66 ± 0.2	0.65 ± 0.2	0.64 ± 0.2	0.56 ± 0.3	0.71 ± 0.2	0.67 ± 0.2	0.67 ± 0.2	0.73 ± 0.2	0.66 ± 0.2
**Number of Interactions between Divalent Cations and Indicated Protein Residues**									
**Glu 118**	N/A^b^	N/A^b^	0	30,306^d^	1140	1261^e^	0	N/A^b^	0
**Asp 123**			0	20,013^d^	18	386^e^	0		0
**Asp 131**			29,851	27,851^d^	786	3204^e^	0		10
**Asp 147**			29,594	29,795^d^	747	2531^e^	0		29,820
**Asp 148**			32,478	30,591^d^	20	153^e^	0		34,203
**Asp 155**			0	30,028^d^	24	691^e^	29,676		0
**Total**			95,239	235,266^d^	4009	13,342^e^	30,467		66,740

a Cα atoms

b Divalent cation interactions with the protein were not applicable (N/A).

c An MD simulation with MCU-NTD_WT_ at 320 K exhibited 74,139 total protein interactions with 1 Ca^2+^ ion; Asp 131 (9536), Asp 147 (28,398) and Asp 148 (31,903) were the primary contact points.

d Interactions between each Ca^2+^ ion and specified residues or the entire protein (total). Ca^2+^ ion 1: Glu 118 (0), Asp 123 (0), Asp 131 (27,850), Asp 147 (29,791), Asp 148 (30,590), Asp 155 (0), total interactions (89,318) Ca^2+^ ion 2: Glu 118 (0), Asp 123 (0), Asp 131 (0), Asp 147 (0), Asp 148 (0), Asp 155 (30,027), total interactions (30,822) Ca^2+^ ion 3: Glu 118 (2), Asp 123 (20,001), Asp 131 (1), Asp 147 (4), Asp 148 (1), Asp 155 (1), total interactions (21,058) Ca^2+^ ion 4: Glu 118 (30,296), Asp 123 (12), Asp 131 (0), Asp 147 (0), Asp 148 (0), Asp 155 (0), total interactions (61,743) Ca^2+^ ion 5: Glu 118 (8), Asp 123 (0), Asp 131 (0), Asp 147 (0), Asp 148 (0), Asp 155 (0), total interactions (32,325)

e Interactions between each Mg^2+^ ion and specified residues or the entire protein (total). Mg^2+^ ion 1: Glu 118 (53), Asp 123 (40), Asp 131 (1015), Asp 147 (708), Asp 148 (29), Asp 155 (210), total interactions (3188) Mg^2+^ ion 2: Glu 118 (221), Asp 123 (79), Asp 131 (861), Asp 147 (586), Asp 148 (12), Asp 155 (145), total interactions (2935) Mg^2+^ ion 3: Glu 118 (473), Asp 123 (54), Asp 131 (365), Asp 147 (389), Asp 148 (29), Asp 155 (97), total interactions (2338) Mg^2+^ ion 4: Glu 118 (355), Asp 123 (61), Asp 131 (475), Asp 147 (462), Asp 148 (55), Asp 155 (156), total interactions (2585) Mg^2+^ ion 5: Glu 118 (159), Asp 123 (152), Asp 131 (488), Asp 147 (386), Asp 148 (28), Asp 155 (83), total interactions (2296)

**Table 3:** Extracted MD simulation parameters for all MCUb-NTD systems obtained after 1 *μs*.

	**Temperature**		**Divalent Cation Concentration**				**Helix Mutations**		**MRAP Mutation**	**Sector Mutation**	
	**MCUb** **310 K**	**MCUb** **320 K**	**MCUb** **+ 1 Ca**^**2+**^	**MCUb** **+ 5 Ca**^**2+**^	**MCUb** **+ 1 Mg**^**2+**^	**MCUb** **+ 5 Mg**^**2+**^	**MCUb** **D99A/E103A**	**MCUb** **D99A/E103A** **+ 1 Ca**^**2+**^	**MCUb** **D116A/D133A** **+ 1 Ca**^**2+**^	**MCUb** **M119R**	**MCU** **M119R** **+ 1 Ca**^**2+**^
**RMSD –** **Total Protein (Å)**^a^	1.9 ± 0.2	2.1 ± 0.3	2.2 ± 0.3	1.8 ± 0.3	2.2 ± 0.2	2.2 ± 0.3	1.8 ± 0.2	2.1 ± 0.3	1.7 ± 0.2	2.0 ± 0.2	1.9 ± 0.2
**RMSD –** **α-helix 1 (Å)**^a^	0.8 ± 0.3	1.2 ± 0.6	1.2 ± 0.5	0.9 ± 0.3	0.9 ± 0.3	1.2 ± 0.5	0.4 ± 0.1	0.5 ± 0.2	0.8 ± 0.2	1.1 ± 0.5	0.9 ± 0.3
**Residue-based S**^**2**^**Order Parameter**	0.65 ± 0.2	0.62 ± 0.2	0.66 ± 0.2	0.65 ± 0.2	0.68 ± 0.2	0.67 ± 0.2	0.74 ± 0.2	0.66 ± 0.2	0.74 ± 0.1	0.65 ± 0.2	0.72 ± 0.1
**Number of Interactions between Divalent Cations and Indicated Protein Residues**											
**Asp 99**	N/A^b^	N/A^b^	29,890	29,929^c^	1918	5668^d^	N/A^b^	0	30,047	N/A^b^	0
**Glu 103**			30,653	30,257^c^	2077	6065^d^		0	29,527		0
**Asp 116**			0	29,887^c^	258	1230^d^		30,186	0		0
**Asp 133**			0	16,002^c^	237	2324^d^		2	0		28,077
**Total**			62,348	206,710^c^	6496	28,667^d^		34,818	61,706		70,381^e^

a Cα atoms

b Divalent cation interactions with the protein were not applicable (N/A).

c Interactions between each Ca^2+^ ion and specified residues or the entire protein (total). Ca^2+^ ion 1: Asp 99 (29,929), Glu 103 (30,257), Asp 116 (0), Asp 133 (0), total interactions (61,022) Ca^2+^ ion 2: Asp 99 (0), Glu 103 (0), Asp 116 (29,872), Asp 133 (0), total interactions (33,822) Ca^2+^ ion 3: Asp 99 (0), Glu 103 (0), Asp 116 (0), Asp 133 (3), total interactions (48,258) Ca^2+^ ion 4: Asp 99 (0), Glu 103 (0), Asp 116 (4), Asp 133 (15,999), total interactions (42,305) Ca^2+^ ion 5: Asp 99 (0), Glu 103 (0), Asp 116 (11), Asp 133 (0), total interactions (21,303)

d Interactions between each Mg^2+^ ion and specified residues or the entire protein (total). Mg^2+^ ion 1: Asp 99 (948), Glu 103 (863), Asp 116 (320), Asp 133 (508), total interactions (5087) Mg^2+^ ion 2: Asp 99 (2917), Glu 103 (3322), Asp 116 (74), Asp 133 (346), total interactions (9117) Mg^2+^ ion 3: Asp 99 (373), Glu 103 (525), Asp 116 (255), Asp 133 (63), total interactions (2328) Mg^2+^ ion 4: Asp 99 (711), Glu 103 (629), Asp 116 (335), Asp 133 (865), total interactions (6746) Mg^2+^ ion 5: Asp 99 (719), Glu 103 (726), Asp 116 (246), Asp 133 (542), total interactions (5389)

e Interactions between the Ca^2+^ ion and the entire protein (total) are predominantly mediated by Asp 133 (28,077 interactions) and Asp 144 (28,375 interactions).

Supplementary material related to this article can be found online at doi:10.1016/j.csbj.2024.12.007.

The following is the Supplementary material related to this article Video S1.

Video S1: MD of MCU-NTD at 310 K.

The following is the Supplementary material related to this article Video S2.

Video S2: MD of MCU-NTD at 320 K.

The following is the Supplementary material related to this article Video S3.

Video S3: MD of MCUb-NTD at 310 K.

The following is the Supplementary material related to this article Video S4.

Video S4: MD of MCUb-NTD at 320 K.

Next, we calculated order parameters (S^2^) as a measure of motion restriction of the backbone N-H bonds [55], [56]. Generally, high S^2^ are expected in structured regions with low internal motion [57]. Indeed, the central helix of MCU-NTD and MCUb-NTD at 310 K (∼37 °C) both have high average S^2^ of 0.67 and 0.72, respectively (Fig. 2E-2G). In-line with the observed RMSD, higher temperature increased S^2^ for the MCU-NTD central helix to 0.72 and decreased S^2^ for the MCUb-NTD central helix to 0.58 (Fig. 2E-2G). By contrast, low S^2^ are expected in regions with high internal motion [57]. Residues in the loops were found to have low S^2^ overall for both MCU-NTD and MCUb-NTD (Fig. 2E-2G). Interestingly, the rigidity of the N-H backbone bonds in L5 of MCUb-NTD was greater than in MCU-NTD at 310 K (∼37 °C) and 320 K (∼47 °C), although there was a greater net increase in rigidification of L5 for MCU-NTD between 310 K (∼37 °C) to 320 K (∼47 °C) (Fig. 2E-2G).

Collectively, the data suggest that an ∼10 °C increase in temperature favours MCU-NTD structural stability and decreased dynamics in contrast to MCUb-NTD, which is destabilized by the higher temperature. This contrasting behaviour may ensure and enhance Ca^2+^ uptake necessary for high ATP production during respiratory burst while tempering MCUb inhibitory function.

### 3.3 MCU-NTD preferentially binds Ca^2+^ and Mg^2+^ in the MRAP region

**Fig. 3:**
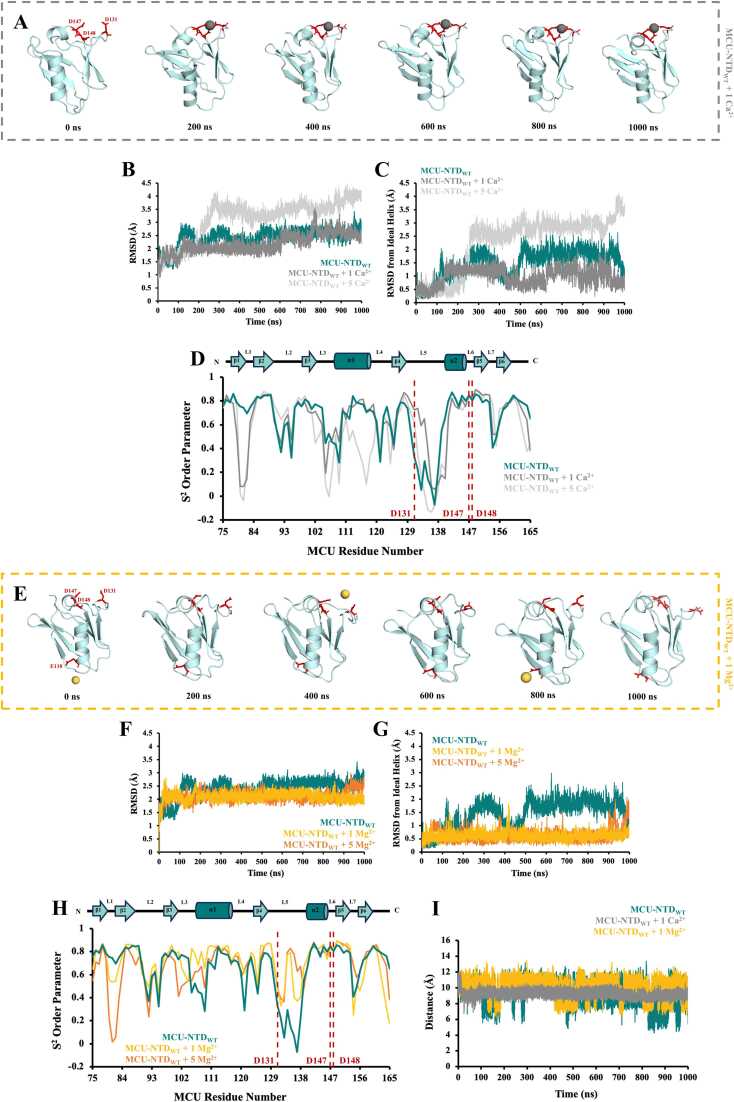
Preferential divalent cation coordination of MCU-NTD in the MRAP region. (A) Snapshots of MCU-NTD_WT_ (teal) taken every 200 ns from the 1 *μs* simulation with 1 Ca^2+^ ion added into the system. Residues D131, D147 and D148 are shown as sticks in red, with the Ca^2+^ ion shown as gray spheres. The average (B) total backbone root-mean-square deviation (RMSD) and (C) central helix RMSD from an ideal helix of MCU-NTD_WT_ were calculated in the absence and presence of 1 and 5 Ca^2+^ ions. (D) Order parameters (S^2^) were calculated as a measure of motion restriction for the backbone N-H bonds of MCU-NTD_WT_ in the absence and presence of 1 and 5 Ca^2+^ ions, with MRAP residues D131, D147 and D148 highlighted with a red dashed line. A schematic diagram indicating the localization of the secondary structure in MCU-NTD_WT_ is shown above the S^2^ order parameter plot. In (*B-D*), MCU-NTD_WT_ in the absence of Ca^2+^ is shown in teal, with 1 Ca^2+^ ion shown in gray and 5 Ca^2+^ ions added shown in light gray. (E) Snapshots of MCU-NTD_WT_ (teal) taken every 200 ns from the 1 *μs* simulation with 1 Mg^2+^ ion added into the system. Residues E118, D131, D147 and D148 are shown as sticks in red, with the Mg^2+^ ion shown as yellow spheres. The average (F) total backbone and (G) central helix RMSDs from an ideal helix of MCU-NTD_WT_ were calculated in the absence and presence of 1 and 5 Mg^2+^ ions. (H) Order parameters (S^2^) were calculated as a measure of motion restriction for the backbone N-H bonds of MCU-NTD_WT_ in the absence and presence of 1 and 5 Mg^2+^ ions, with MRAP residues D131, D147 and D148 highlighted with a red dashed line and the schematic above indicating localization of secondary structure. In (*F-H*), MCU-NTD_WT_ in the absence of Mg^2+^ is shown in teal, with 1 Mg^2+^ ion shown in yellow and 5 Mg^2+^ ions added shown in orange. (I) Distance fluctuations between the MRAP D131 and D148 Oδ1 atoms were measured in MCU-NTD_WT_ in the absence (teal) and presence of 1 Ca^2+^ (gray) or 1 Mg^2+^ ion (yellow). A two-sample F-test for variances indicates smaller fluctuations in the presence of both divalent cations, with Ca^2+^ exhibiting the smallest fluctuations.

The MCU-NTD was previously shown to bind Ca^2+^ and Mg^2+^ through a cluster of negatively charged residues forming the MRAP [29]. Thus, we next evaluated the effects of divalent cation coordination on stability and dynamics of wild-type MCU-NTD (MCU-NTD_WT_). First, we included 1 Ca^2+^ ion in our MCU-NTD_WT_ system at 310 K (∼37 °C). Over the 1 *μs* simulation time, this single Ca^2+^ ion underwent 95,239 total interactions with MCU-NTD_WT_, with ∼97 % contacts occurring with MRAP residues D131, D147 and D148 (i.e. 29,851, 29,594 and 32,478 interactions, respectively) (Fig. 3A, Table 2, Fig. S1A and Video S5). Note that number of interactions were taken as the sum of the ≤ 4 Å distances over 1 *μs* in 100 ps increments between any atom on the residue of interest and the divalent cation. When 5 Ca^2+^ ions were added into the MCU-NTD_WT_ system, the same single Ca^2+^ ion remained bound to MRAP, while three of the additional Ca^2+^ ions predominantly interacted with E118, D123 and D155 (Table 2, Fig. S1A and S1C and Video S6). Interestingly, the interactions of Ca^2+^ with E118 destabilized the central helix, increasing the average total backbone Cα and central helix RMSDs by ∼0.9 Å and ∼1.3 Å, respectively (Figs. 3B and 3C, Table 2, Fig. S1C and Video S6). Note that the central helix RMSD reported is relative to ideal helix geometry. S^2^ for D147 and D148 of MRAP remained high at ∼0.8, independent of divalent cations (Fig. 3D). However, the D131 S^2^ markedly increased from 0.33 to 0.62 and 0.72 upon addition of 5 Ca^2+^ ions and 1 Ca^2+^ ion, respectively, indicative of a restriction of the N-H bond and suppressed dynamics on the ps timescale (Figs. 3D and 3E). Interestingly, at increased temperature (∼47 ˚C) where MCU-NTD_WT_ was more structurally stable, the dynamics of D131 remained high (*i.e.* S^2^ of 0.38) in the presence of 1 Ca^2+^ ion, supporting reduced contacts between D131 and Ca^2+^ compared to D147 and D148 of MRAP (Fig. S2, Table 2 and Video S7).

Supplementary material related to this article can be found online at doi:10.1016/j.csbj.2024.12.007.

The following is the Supplementary material related to this article Video S5.

Video S5: MD of MCU-NTD with 1 Ca^2+^ at 310 K.

The following is the Supplementary material related to this article Video S6.

Video S6: MD of MCU-NTD with 5 Ca^2+^ at 310 K.

The following is the Supplementary material related to this article Video S7.

Video S7: MD of MCU-NTD with 1 Ca^2+^ at 320 K.

In contrast, a single Mg^2+^ ion in the MCU-NTD_WT_ system showed only 4009 total Mg^2+^:protein interactions, with most of the contacts identified with D131 and D147 (*i.e.* ∼20 % and ∼19 %, respectively; ∼39 % of the total count) of MRAP. Nevertheless, additional interactions occurred with E118 (*i.e.* ∼28 % of the total) of the central helix as the ion hopped from the helix to MRAP over the 1 *μs* simulation time (Fig. 3E, Table 2, Fig. S1B and S1D, Video S8). Adding 5 Mg^2+^ ions to the system dramatically increased Mg^2+^ contacts with MRAP D131 and D147 (*i.e.* ∼3–4-fold) but hardly altered contacts with E118 (Video S9). The presence of 1 or 5 Mg^2+^ ions decreased the average total backbone Cα RMSD and central helix RMSD (*i.e.* relative to an ideal helix) where E118 is situated (Figs. 3F, 3G and Table 2). Mg^2+^ also decreased the dynamics of D131 as S^2^ increased from 0.33 to 0.51 and 0.48 with the addition of 1 Mg^2+^ ion and 5 Mg^2+^ ions, respectively (Fig. 3H).

Supplementary material related to this article can be found online at doi:10.1016/j.csbj.2024.12.007.

The following is the Supplementary material related to this article Video S8.

Video S8: MD of MCU-NTD with 1 Mg^2+^ at 310 K.

The following is the Supplementary material related to this article Video S9.

Video S9: MD of MCU-NTD with 5 Mg^2+^ at 310 K.

Collectively, these MCU-NTD_WT_ MD data suggest that single Ca^2+^ binding occurs preferentially in MRAP, increasing L5 rigidity, while additional Ca^2+^ ions interact with the central helix, destabilizing the structure. In contrast, a single Mg^2+^ interacts with both the central helix and MRAP, increasing L5 rigidity and enhancing structural stability, while additional Mg^2+^ increases the occupancy in MRAP. Congruent with more stable Ca^2+^ versus Mg^2+^ interactions within MRAP, D131 and D148 Oδ1 atom distances show smaller fluctuations in the presenc of Ca^2+^ over the 1 *μs* simulation (Fig. 3I).

### 3.4 MCUb-NTD preferentially binds Ca^2+^ and Mg^2+^ at the central helix

**Fig. 4:**
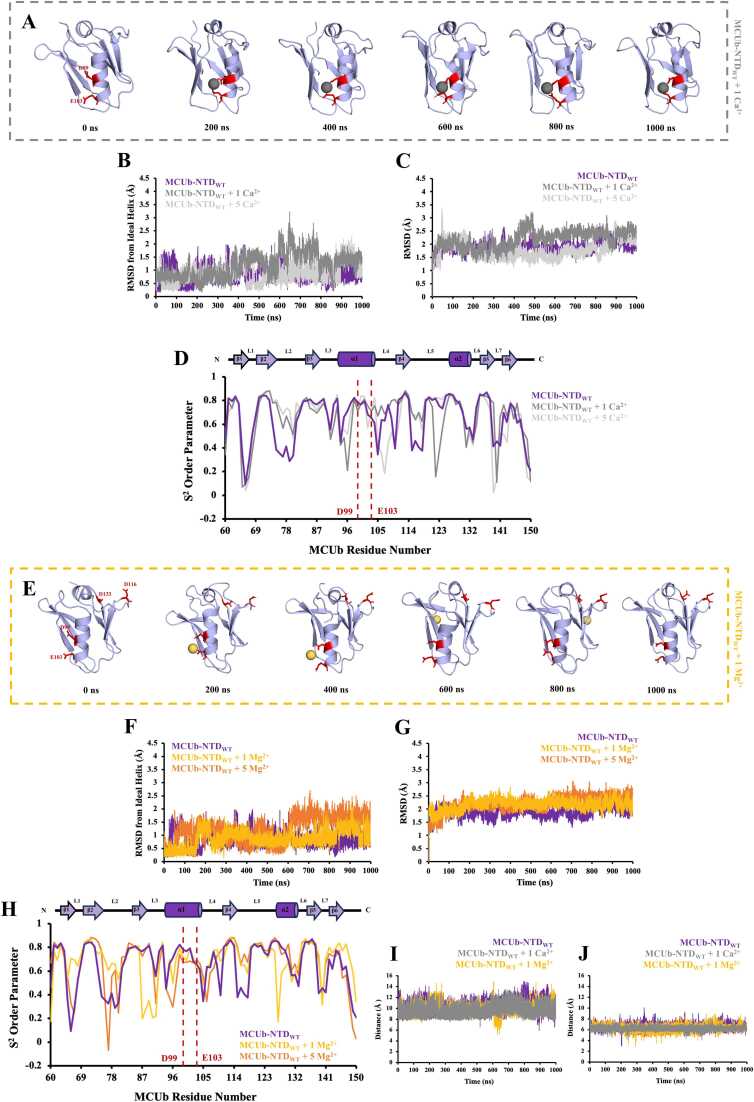
Preferential divalent cation coordination of MCUb-NTD at the central helix. (A) Snapshots of MCUb-NTD_WT_ (purple) were taken every 200 ns from the 1 *μs* simulation with 1 Ca^2+^ ion added into the system. Residues D99 and E103 are shown as sticks in red, with the Ca^2+^ ion shown as gray spheres. The average (B) central helix root-mean-square deviation (RMSD) from an ideal helix and (C) total backbone RMSD of MCUb-NTD_WT_ were calculated in the absence and presence of 1 and 5 Ca^2+^ ions. (D) Order parameters (S^2^) were calculated as a measure of motion restriction for the backbone N-H bonds of MCUb-NTD_WT_ in the absence and presence of 1 and 5 Ca^2+^ ions, with D99 and E103 highlighted with a red dashed line. A schematic diagram indicating the localization of the secondary structure in MCUb-NTD_WT_ is shown above the S^2^ order parameter plot. In (*B-D*), MCUb-NTD_WT_ in the absence of Ca^2+^ is shown in purple, with 1 Ca^2+^ ion shown in gray and 5 Ca^2+^ ions added shown in light gray. (E) Snapshots of MCUb-NTD_WT_ (purple) taken every 200 ns from the 1 *μs* simulation with 1 Mg^2+^ ion added into the system. Residues D99, E103, D116 and D133 are shown as sticks in red, with the Mg^2+^ ion shown as yellow spheres. The average (F) central helix RMSD from an ideal helix and (G) total backbone RMSD of MCUb-NTD_WT_ were calculated in the absence and presence of 1 and 5 Mg^2+^ ions. (H) Order parameters (S^2^) were calculated as a measure of motion restriction for the backbone N-H bonds of MCUb-NTD_WT_ in the absence and presence of 1 and 5 Mg^2+^ ions, with residues D99 and E103 highlighted with a red dashed line and the schematic above indicating localization of secondary structure. In (*F-H*), MCUb-NTD_WT_ in the absence of Mg^2+^ is shown in purple, with 1 Mg^2+^ ion shown in yellow and 5 Mg^2+^ ions added shown in orange. (I) Distance fluctuations between the MCUb-NTD_WT_ corresponding MRAP residues D116 and D133 Oδ1 atoms, as well as (J) distance fluctuations between the central helix residues D99 and E103 Oδ1 atoms, were measured in the absence (purple) and presence of 1 Ca^2+^ (gray) or 1 Mg^2+^ ion (yellow). A two-sample F-test for variances indicates smaller distance fluctuations between D99 and E103 in the presence of Ca^2+^.

We next repeated the simulations with our wild-type MCUb-NTD homology model (MCUb-NTD_WT_). Remarkably, when 1 Ca^2+^ ion was added into the MCUb-NTD_WT_ system, instead of interacting with the MRAP residues akin to MCU, the Ca^2+^ ion interacted with D99 and E103 of the central helix, showing 29,890 and 30,653 interactions, respectively (Fig. 4A, Table 3, Fig. S1E and Video S10). When 5 Ca^2+^ ions were added into the MCUb-NTD_WT_ system, one Ca^2+^ remained contacting D99 and E103, while two of the additional Ca^2+^ ions predominantly interacted with D116 and D133 of MRAP in MCUb (Fig. S1E and S1G and Video S11). Sole Ca^2+^ binding to D99 and E103 perturbed the central helix ideality, increasing RMSD by ∼0.4 Å (Fig. 4B, Table 3). Nevertheless, upon additional Ca^2+^ interactions with the MRAP region, the domain structure was stabilized with both total backbone Cα and central helix RMSDs decreasing by ∼0.4 and ∼0.3 Å, respectively, compared to the presence of the single Ca^2+^ ion (Figs. 4B and 4C, Table 3). Indeed, average S^2^ in the central helix decreased with 1 Ca^2+^ ion and 5 Ca^2+^ ions (*i.e.* became more mobile) but retained more rigidity with 5 Ca^2+^ ions (Fig. 4D).

Supplementary material related to this article can be found online at doi:10.1016/j.csbj.2024.12.007.

The following is the Supplementary material related to this article Video S10.

Video S10: MD of MCUb-NTD with 1 Ca^2+^ at 310 K.

The following is the Supplementary material related to this article Video S11.

Video S11: MD of MCUb-NTD with 5 Ca^2+^ at 310 K.

When 1 or 5 Mg^2+^ ions were added into the MCUb-NTD_WT_ system, Mg^2+^ predominantly interacted with D99 and E103 of the central helix (*i.e.* ∼61 % of all contacts) but also showed a small percentage of interactions with the D116 and D133 MRAP residues (*i.e.* ∼4 % each; ∼8 % of total) (Fig. 4E, Table 3, Fig. S1F and S1H, Video S12, Video S13). Notably, Mg^2+^ destabilized the MCUb-NTD_WT_ central helix and overall backbone fold as RMSDs were elevated relative to the control no divalent cation simulation (*i.e.* by ∼0.1 and ∼0.3 Å, respectively, in the 1 Mg^2+^ ion simulation; by ∼0.4 and 0.3 Å, respectively, in the 5 Mg^2+^ ion simulation) (Figs. 4F and 4G, Table 3). Congruently, 1 and 5 Mg^2+^ ions incrementally lowered S^2^ of the central helix (Fig. 4H). Thus, single Ca^2+^ ion binding exclusively occurs with D99 and E103 of the central helix of MCUb-NTD_WT_, destabilizing the structure, while additional Ca^2+^ interactions occur in the MRAP region, re-stabilizing the domain; moreover, Mg^2+^ interactions also preferentially occur with D99 and E103 of the central helix, but additional contacts are observed with MRAP when single or multiple Mg^2+^ ions are included in the system.

Supplementary material related to this article can be found online at doi:10.1016/j.csbj.2024.12.007.

The following is the Supplementary material related to this article Video S12.

Video S12: MD of MCUb-NTD with 1 Mg^2+^ at 310 K.

The following is the Supplementary material related to this article Video S13.

Video S13: MD of MCUb-NTD with 5 Mg^2+^ at 310 K.

Overall, the MD data suggest that MCUb-NTD preferentially interacts with divalent cations via the negatively charged residues on the central helix in contrast to MCU-NTD, which favours binding of these cations on MRAP. Remarkably, the MCU MRAP residues are not fully conserved in MCUb while the ion coordinating residues of the MCUb central helix are not fully conserved in MCU. Interestingly, the average distances between the central helix D99 and E103 Oδ1 atoms of the central helix are ∼3 Å smaller than the D133 and D148 Oδ1 atoms of the MCU-NTD MRAP in the presence of the divalent cations (Figs. 4I and 4J).

### 3.5 MRAP mutations disrupt divalent cation coordination in MCU-NTD but not MCUb-NTD

**Fig. 5:**
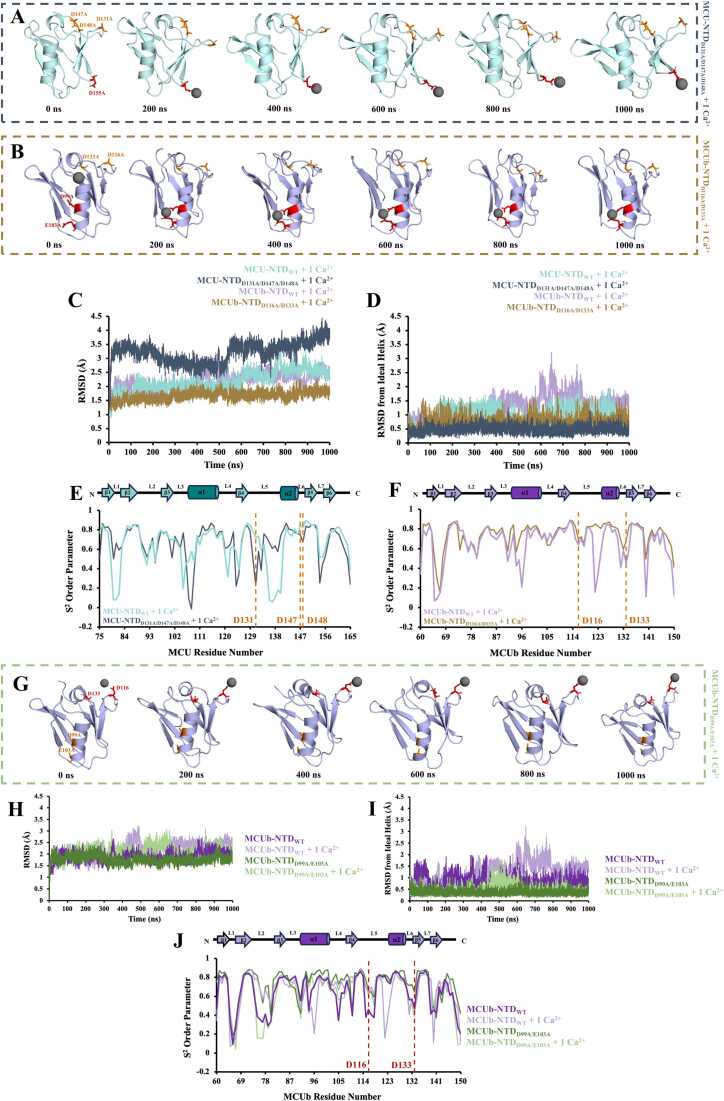
Mutation to the distinct divalent cation coordinating residues on MCU-NTD and MCUb-NTD. Snapshots were taken every 200 ns from the 1 *μs* simulation with 1 Ca^2+^ ion added into the (A) MCU-NTD_D131A/D147A/D148A_ (teal) system and (B) MCUb-NTD_D116A/D133A_ (purple). In (*A*) residue D155 is shown as sticks in red. In (*B*) residues D99 and E103 on the central helix are shown as sticks in red. In (*A-B*) the mutated MRAP residues (*i.e.* residues D131, D147, and D148 in MCU conserved as D116 and D133 in MCUb) are shown as sticks in orange and the Ca^2+^ ion is shown with gray spheres. (C) The average root-mean-square deviation (RMSD) of the 92 Cα atoms for MCU-NTD and MCUb-NTD, wild-type and MRAP variants, in the presence of 1 Ca^2+^ ion was determined, along with the (D) RMSD of the central helix from an ideal geometry. (E) Order parameters (S^2^) were calculated as a measure of motion restriction for the backbone N-H bonds of MCU-NTD, wild-type and MRAP mutated, in the presence of 1 Ca^2+^ ion, with residues D131, D147 and D148 highlighted with an orange dashed line, along with the schematic above indicating localization of secondary structure. In (*C-E*), MCU-NTD_WT_ in the presence of Ca^2+^ is shown in light teal, with MCU-NTD_D131A/D147A/D148A_ in the presence of Ca^2+^ shown in dark blue. (F) Order parameters (S^2^) were calculated as a measure of motion restriction for the backbone N-H bonds of MCUb-NTD, wild-type and MRAP mutated, in the presence of 1 Ca^2+^ ion, with residues D116 and D133 highlighted with an orange dashed line, along with the schematic above indicating localization of secondary structure. In (*C,D,F*), MCUb-NTD_WT_ in the presence of Ca^2+^ is shown in light purple, with MCUb-NTD_D116A/D133A_ in the presence of Ca^2+^ shown in brown. (G) Snapshots of MCUb-NTD_D99A/E103A_ (purple) were taken every 200 ns from the 1 *μs* simulation with 1 Ca^2+^ ion added into the system, shown as gray spheres. Mutated residues D99 and E103 are shown as sticks in orange and residues D116 and D133 are shown as sticks in red. (H) The average RMSD of the 92 Cα atoms for MCUb-NTD, wild-type and D99A/E103A variants, in the absence and presence of 1 Ca^2+^ ion was determined, along with the (I) RMSD of the central helix from an ideal geometry. (J) Order parameters (S^2^) were calculated as a measure of motion restriction for the backbone N-H bonds of MCUb-NTD_D99A/E103A_ compared to wild-type in the absence and presence of 1 Ca^2+^ ion, with residues D116 and D133 highlighted with a red dashed line, along with the schematic above indicating localization of secondary structure. In (H-J), in the absence of divalent cations, MCUb-NTD_WT_ is shown in purple, MCUb-NTD_D99A/E103A_ is shown in dark green, and in the presence of 1 Ca^2+^ ion, MCUb-NTD_WT_ is shown in light purple and MCUb-NTD_D99A/E103A_ is shown in light green.

To reinforce that the dominant divalent cation coordinating residues on the MCUb-NTD_WT_ were distinct from MRAP, we next mutated the three MRAP residues in MCU-NTD (*i.e.* D131A, D147A, and D148A) and the two conserved MRAP residues in MCUb-NTD (*i.e*. D116A and D133A). As expected, when 1 Ca^2+^ ion was added into the MCU-NTD_D131A/D147A/D148A_ system, Ca^2+^ did not interact with the MRAP region but rather showed 29,676 interactions with D155 in loop 7 (L7) (Fig. 5A, Table 2, Video S14). In contrast, when 1 Ca^2+^ ion was added into the MCUb-NTD_D116A/D133A_ system, the Ca^2+^ showed 30,047 and 29,527 interactions with D99 and E103, respectively, remarkably similar to our observations with MCUb-NTD_WT_ (Fig. 5B, Table 3, Video S15). Similar to additional Ca^2+^ interactions (*i.e.* 5 Ca^2+^ ions added) shown to destabilize the MCU-NTD (Figs. 3B and 3C, Table 2), mutation of the MRAP residues increased the total backbone Cα RMSD for MCU-NTD_D131A/D147A/D148A_ (*i.e.* by ∼1.0 Å) (Fig. 5C and Table 2). Conversely, the total backbone Cα RMSD for MCUb-NTD_D116A/D133A_ decreased (*i.e.* by ∼0.4 Å) (Fig. 5C and Table 3), similar to additional Ca^2+^ interactions with the MRAP region shown to stabilize the MCUb-NTD. Both MCU-NTD_D131A/D147A/D148A_ and MCUb-NTD_D116A/D133A_ promoted more ideal central helix geometry, lowering the RMSD by ∼0.5 and ∼0.4 Å, respectively, compared to the WT simulations with 1 Ca^2+^ ion (Fig. 5D, Table 2, Table 3). However, the central helix S^2^ minimally decreased for MCU-NTD_D131A/D147A/D148A_, given that the 1 Ca^2+^ ion predominantly interacted with D155 of L7 (Figs. 5A and 5E, Table 2). Further, D131A, D147A and D148A S^2^ values were lower for the MCU-NTD_D131A/D147A/D148A_ compared to MCU-NTD_WT_ in the presence of 1 Ca^2+^, indicating increased MRAP backbone dynamics (Fig. 5E). On the other hand, the central helix S^2^ increased for MCUb-NTD_D116A/D133A_, with D116A and D133A S^2^ increased for MCUb-NTD_D116A/D133A_ compared to MCUb-NTD_WT_, consistent with the predominant Ca^2+^ interactions with the central helix of both MCUb-NTD_D116A/D133A_ and MCUb-NTD_WT_ (Fig. 5F, Table 3).

Supplementary material related to this article can be found online at doi:10.1016/j.csbj.2024.12.007.

The following is the Supplementary material related to this article Video S14.

Video S14: MD of MCU-NTD MRAP triple mutant (D131A/D147A/D148A) with 1 Ca^2+^ at 310 K.

The following is the Supplementary material related to this article Video S15.

Video S15: MD of MCUb-NTD D116A/D133A with 1 Ca^2+^ at 310 K.

### 3.6 MCUb-NTD central helix mutations switch divalent cation coordination to MRAP

Given mutation of the conserved MRAP residues in MCUb-NTD_D116A/D133A_ did not alter Ca^2+^ coordination, we next varied the divalent cation coordinating residues on the central helix of MCUb-NTD (*i.e*. D99A and E103A), creating MCUb-NTD_D99A/E103A_. The MCUb-NTD_D99A/E103A_ double mutation caused a switch in the coordination site of the single Ca^2+^ ion from the central helix to D116, one of the conserved MRAP residues in MCUb, showing 30,186 interactions over 1 *μs* (Fig. 5G, Table 3, Video S16 and S17). The D99A/E103A double mutation had a negligible effect on the total backbone Cα RMSD compared to MCUb-NTD_WT_ in the absence or presence of 1 Ca^2+^ ion (Fig. 5H and Table 3); however, the central helix in MCUb-NTD_D99A/E103A_ more closely resembled an ideal helix compared to MCUb-NTD_WT_ in the absence or presence of Ca^2+^, perhaps due to the enrichment in Ala [58] (Fig. 5I and Table 3). Further, the central helix S^2^ increased in either the absence or presence of Ca^2+^ compared to MCUb-NTD_WT_ (*i.e.* became more rigid). Notably, S^2^ of the conserved MCUb-NTD_D99A/E103A_ MRAP residues (*i.e*. D116 and D133) in the absence of Ca^2+^ increased considerably, indicating less internal motion (Fig. 5J). However, with 1 Ca^2+^ ion added into the system, S^2^ of D133 decreased from 0.45 to 0.39 while D116 remained high, only marginally changing from 0.64 to 0.62, supporting the predominate interactions identified between the Ca^2+^ ion and D116 of MCUb-NTD_D99A/E103A_ over the simulation time (Figs. 5G and 5J, Table 3**,** Video S17).

Supplementary material related to this article can be found online at doi:10.1016/j.csbj.2024.12.007.

The following is the Supplementary material related to this article Video S16.

Video S16: MD of MCUb-NTD D99A/E103A at 310 K.

The following is the Supplementary material related to this article Video S17.

Video S17: MD of MCUb-NTD D99A/E103A with 1 Ca^2+^ at 310 K.

Collectively, our mutant MD data reinforce that MCU- and MCUb-NTDs have distinctly preferred Ca^2+^ coordination sites, with the MRAP region binding the divalent cation in MCU and the central helix being the primary interaction site of the ion in MCUb.

### 3.7 Both MCU-NTD and MCUb-NTD show one analogous sector of co-evolving residues

**Fig. 6:**
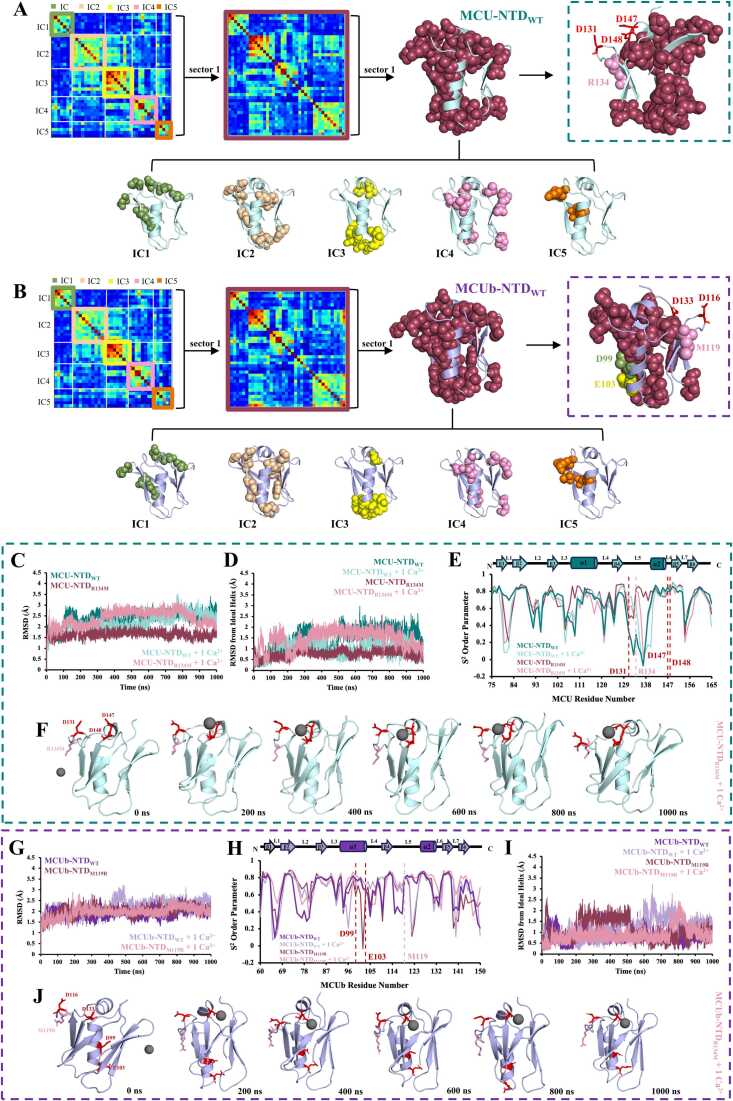
pySCA identification of a co-evolving sector of residues within the MCU-NTD and MCUb-NTD. (A) On the left, the decomposed statistical coupling analysis (SCA) matrix of the residues making up the independent components (ICs) of MCU-NTD, with the 40 residues identified to co-evolve as one sector, shown by the subsequent matrix. The 40 residues making up the single sector of co-evolving residues for the MCU-NTD (teal) were mapped on the refined crystal structure (8URG.pdb) as spheres in maroon, with the residues of each IC shown below (IC1, green; IC2, beige; IC3, yellow; IC4, pink; IC5, orange). Highlighted in a box on the right is MCU-NTD (teal), with the co-evolving sector of residues shown as spheres in maroon, R134 within the sector highlighted in pink spheres, and MRAP residues not part of the co-evolving sector shown as sticks in red. (B) On the left, the decomposed statistical coupling analysis (SCA) matrix of the residues making up the independent components (ICs) of MCUb-NTD, with the 38 residues identified to co-evolve as one sector, shown by the subsequent matrix. The 38 residues making up the single sector of co-evolving residues for the MCUb-NTD (purple) were mapped on the homology modeled structure as spheres in maroon, with the residues of each IC shown below (IC1, green; IC2, beige; IC3, yellow; IC4, pink; IC5, orange). Highlighted in a box on the right is MCUb-NTD (purple), with the co-evolving sector of residues shown as spheres in maroon, M119 highlighted in pink spheres, D99 shown in green and E103 shown in yellow. (C) The average root-mean-square deviation (RMSD) of the 92Cα atoms for MCU-NTD, wild-type and R134M variants, in the absence and presence of Ca^2+^ was determined, along with the (D) RMSD of the central helix from an ideal geometry. (E) Order parameters (S^2^) were calculated as a measure of motion restriction for the backbone N-H bonds of MCU-NTD, wild-type and R134M variants, in the absence and presence of Ca^2+^, with residues R134 highlighted with a pink dashed line and MRAP residues D131, D147 and D148 highlighted with a red dashed line, along with the schematic above indicating localization of secondary structure. In (*C-E*), in the absence of divalent cations, MCU-NTD_WT_ is shown in teal, MCU-NTD_R134M_ is shown in maroon, and in the presence of 1 Ca^2+^ ion, MCU-NTD_WT_ is shown in light teal and MCU-NTD_R134M_ is shown in light pink. (F) Snapshots of MCU-NTD_R134M_ (teal) were taken every 200 ns from the 1 *μs* simulation with 1 Ca^2+^ ion added into the system, shown as gray spheres. Mutated residue R134M is shown as sticks in pink, with MRAP residues D131, D147 and D148 shown as sticks in red. (G) The average RMSD of the 92Cα atoms for MCUb-NTD, wild-type and M119R variants, in the absence and presence of Ca^2+^ was determined. (H) Order parameters (S^2^) were calculated as a measure of motion restriction for the backbone N-H bonds of MCUb-NTD, wild-type and M119R variants, in the absence and presence of Ca^2+^, with residues M119 highlighted with a pink dashed line and D99 and E103 highlighted with a red dashed line, along with the schematic above indicating localization of secondary structure. (I) RMSD of the central helix from an ideal geometry of MCUb-NTD, wild-type and M119R variants, in the absence and presence of Ca^2+^. In (*G-I*), in the absence of divalent cations, MCUb-NTD_WT_ is shown in purple, MCUb-NTD_M119R_ is shown in maroon, and in the presence of 1 Ca^2+^ ion, MCUb-NTD_WT_ is shown in light purple and MCUb-NTD_M119R_ is shown in light pink. (J) Snapshots of MCUb-NTD_M119R_ (purple) were taken every 200 ns from the 1 *μs* simulation with 1 Ca^2+^ ion added into the system, shown as gray spheres. Mutated residue M119R is shown as sticks in pink, with conserved MRAP residues D116 and D133, along with D99 and E103 shown as sticks in red.

To determine if the distinct divalent cation coordinating mechanisms are connected, python-based statistical coupling analysis (pySCA) was used to identify contiguous networks of co-evolving residues [31]. In pySCA [59], correlations are not solely based on amino acid proximity [32], but rather amino acids are grouped based on residues correlated across orthologues and paralogues (*i.e.* eigenmodes) [60], revealing independent components (ICs) of residue positions co-evolving with one another [31]. From the 91 positions of a multiple sequence alignment of 1948 sequences and 2226 sequences for MCU and MCUb used as the query sequence, respectively, pySCA revealed 40 residues for MCU-NTD and 38 residues for MCUb-NTD, were significantly correlated in 5 ICs each (Fig. 6A and 6B, Table S1). Since each IC was not completely independent (*i.e.* warmer pixels indicating correlated residues observed between ICs), we grouped all ICs into the same larger cluster of co-evolving residues, referred to as a sector (Fig. 6A and 6B). A single sector was identified for both MCU-NTD and MCUb-NTD, likely due to the small domain size and high sequence similarities. Most relevant to the present work, the two Ca^2+^ coordinating residues of the MCUb-NTD central helix (*i.e.* D99 and E103) were identified to correlate between multiple ICs (Fig. 6B). In contrast, MRAP residues were neither identified as part of the sector in MCUb-NTD nor MCU-NTD (Fig. 6A and 6B, Table S1).

We next evaluated the effects of altering the network of co-evolving residues by mutation and MD simulations. The MCU-NTD R134 and the aligned MCUb-NTD M119 position showed the greatest differences in S^2^ (*i.e.* 0.23 and 0.82, respectively) compared to each other in our divalent cation-free simulations. Further, this position was part of the sector for both MCU-NTD and MCUb-NTD. Thus, we swapped the residue type at R134 of MCU-NTD and M119 of MCUb-NTD to the type found in the other paralog (*i.e*. MCU-NTD_R134M_ and MCUb-NTD_M119R_). MCU-NTD_R134M_ in the absence of divalent cations showed reduced total backbone Cα and central helix RMSDs (*i.e.* relative to ideal geometry) compared to MCU-NTD_WT_ over 1 *μs* (Fig. 6C and 6D, Table 2, Video S18). Indeed, the average S^2^ of the domain increased, and specifically, the R134M position increased from 0.29 to 0.83, becoming more similar to MCUb-NTD (Fig. 6E and Table 2). Congruent with MCUb-NTD_WT_, when 1 Ca^2+^ ion was added to the MCU-NTD_R134M_ system, the total backbone Cα RMSD increased while the central helix ideality decreased (Fig. 6 C and 6E, Table 2). Nevertheless, MRAP remained the predominant region of interaction as the Ca^2+^ ion showed 29,820 and 34,203 contacts with D147 and D148 of MCU-NTD_R134M_, respectively, but minimal interactions with D131, indicative of increased dynamics as observed by decreased S^2^ of 131 (Fig. 6E, Table 2, Fig. S1J, Video S19).

Supplementary material related to this article can be found online at doi:10.1016/j.csbj.2024.12.007.

The following is the Supplementary material related to this article Video S18.

Video S18: MD of MCU-NTD R134M at 310 K.

The following is the Supplementary material related to this article Video S19.

Video S19: MD of MCU-NTD R134M with 1 Ca^2+^ at 310 K.

Contrary to MCU-NTD_R134M_, the M119R mutation in MCUb-NTD_M119R_ had negligible effects on the overall backbone Cα RMSD of the domain as well as the S^2^ of residue position 119 (*i.e.* aligned with R134 in MCU-NTD) (Fig. 6G and 6H, Table 3). However, the RMSD of the central helix increased (*i.e.* by ∼0.3 Å) relative to ideal geometry, consistent with residues of this central helix being part of the same sector as M119R despite being on the opposite face of the protein (Fig. 6I, Table 3**,** Video S20). Further, congruent with the exclusion of MRAP residues but inclusion of D99 and E103 in the sector, when 1 Ca^2+^ ion was added into the MCUb-NTD_M119R_ system, interactions between the Ca^2+^ ion and D99 and E103 were abrogated over the simulation time. Instead, the Ca^2+^ ion showed 28,077 interactions with MRAP D133, akin to the MCU-NTD_WT_ that harbors Arg at the aligned MCUb M119R position and natively interacts with Ca^2+^ via the MRAP region (Fig. 6J, Table 3, Video S21). The Ca^2+^ ion, predominantly interacting with D133, slightly decreased the overall backbone Cα and ideal central helix RMSDs by ∼0.1 and 0.2 Å, respectively, similar to the Ca^2+^ ion interacting with MRAP D116 in MCUb-NTD_D99A/E103A_ (Fig. 6G and 6I, Table 3). The S^2^ of D99 and E103 minimally changed with the Ca^2+^ addition, as the divalent cation primarily interacted with the MRAP region (Fig. 6H).

Supplementary material related to this article can be found online at doi:10.1016/j.csbj.2024.12.007.

The following is the Supplementary material related to this article Video S20.

Video S20: MD of MCUb-NTD M119R at 310 K.

The following is the Supplementary material related to this article Video S21.

Video S21: MD of MCUb-NTD M119R with 1 Ca^2+^ at 310 K.

Together, our pySCA and MD analyses suggest that central helix residues of MCUb-NTD involved in divalent cation coordination co-evolved within a sector of residues that does not include MRAP. Remarkably, perturbing the network of co-evolving residues in MCUb-NTD with the M119R mutation abrogates the coordination of Ca^2+^ at the central helix on the opposite face of the protein, shifting the divalent cation interaction to the MRAP region, similar to MCU-NTD_WT_ that natively has Arg at the aligned 134 position. This Ca^2+^ binding site switch in MCUb was also observed when we directly mutated D99 and E103 (see above).

### 3.8 Mutation to either the MCUb-NTD central helix or sector of co-evolving residues negates Ca^2+^-dependent MCUb-NTD structural changes in vitro

**Fig. 7:**
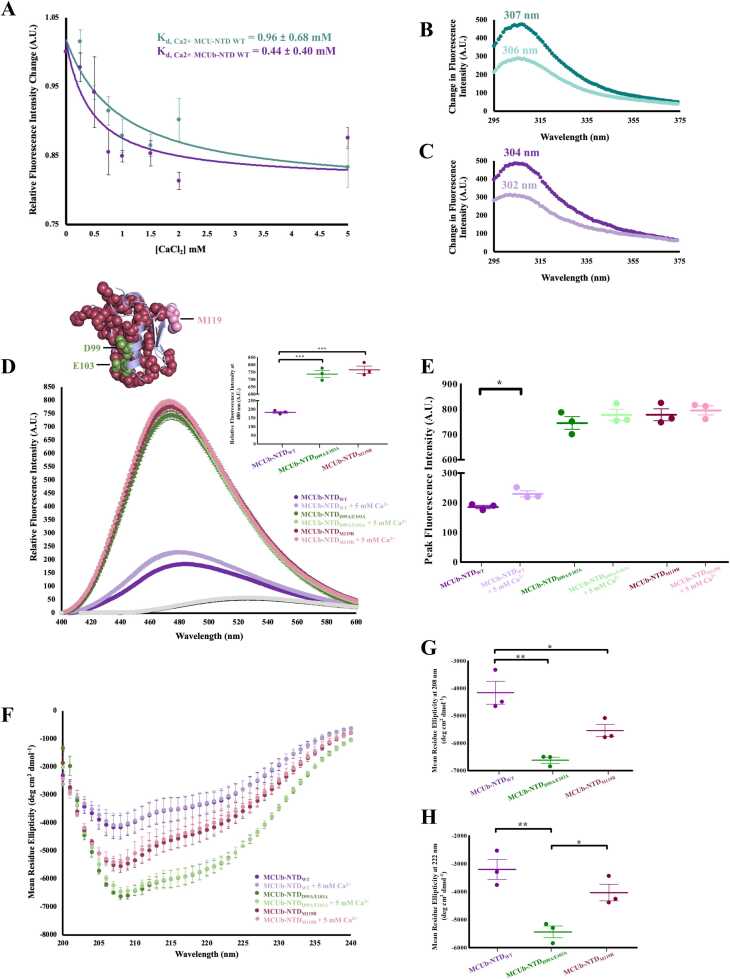
Ca^2+^ binding affinity and structural sensitivity of recombinant MCUb-NTD *in vitro*. (A) MCUb-NTD_WT_ and MCU-NTD_WT_ Ca^2+^ binding curves constructed from changes in maximal intrinsic protein fluorescence. Titrations were performed with increasing concentrations of CaCl_2_ (0–5 mM) at 22.5 ˚C. Data (closed circles) was fit to a one-site binding model that takes into account protein concentration (solid lines). (B) Representative intrinsic fluorescence emission spectra of MCU-NTD_WT_ in the absence (dark teal) and presence (light teal) of 5 mM CaCl_2_. (C) Representative intrinsic fluorescence emission spectra of MCUb-NTD_WT_ in the absence (dark purple) and presence (light purple) of 5 mM CaCl_2_. (D) ANS fluorescence emission spectra of MCUb-NTD_WT,_ MCUb-NTD_D99A/E103A_ and MCUb-NTD_M119R_ in the absence and presence of 5 mM CaCl_2_ with an inset showing the statistical comparison using a one-way ANOVA and Tukey’s HSD post-hoc test of the relative fluorescence intensity at 480 nm in the absence of CaCl_2_ (***P < 0.001). The three-dimensional MCUb-NTD_WT_ structure is shown above the ANS fluorescence emission spectra, highlighting the co-evolving sector of residues identified by pySCA in maroon spheres with M119 (pink) and D99/E103 (green). (E) A paired *t*-test was used to compare the peak fluorescence intensity of MCUb-NTD_WT,_ MCUb-NTD_D99A/E103A_ and MCUb-NTD_M119R_ in the absence and presence of 5 mM CaCl_2_, where *P < 0.05. Extrinsic ANS fluorescence experiments were conducted with 0.25 mg mL^−1^ protein at 25 ˚C. (F) Far-UV CD spectra of MCUb-NTD_WT,_ MCUb-NTD_D99A/E103A_ and MCUb-NTD_M119R_ in the absence and presence of 5 mM CaCl_2_, with statistical comparison using a one-way ANOVA and Tukey’s HSD post-hoc test of the mean residue ellipticity at (G) 208 nm and (H) 222 nm in the absence of CaCl_2_ (**P < 0.01, *P < 0.05). Far-UV CD experiments were conducted with 0.35 mg mL^−1^ protein, with spectra obtained at 20 ˚C. All experiments were conducted in 20 mM Tris (pH 8.5), 150 mM NaCl, and 1 mM DTT. In (*A, D, E, F, G, H*), data are means ± SEM from n = 3 separate experiments. In (*A-C*), data were acquired using an excitation wavelength of 280 nm.

Having observed a sensitivity of Ca^2+^ binding on mutations to the central helix or sector of co-evolving residues *in silico*, we next used purified recombinant human MCUb-NTD to assess the sensitivity of the domain to Ca^2+^ *in vitro*. We first monitored intrinsic fluorescence changes as a function of increasing CaCl_2_ to ascertain the affinity of Ca^2+^ for MCUb-NTD_WT_. The maximal fluorescence emission intensity decreased concomitant with a blue shift in the peak maximum wavelength, consistent with a small structural change associated with the binding. Further, the MCUb-NTD_WT_ Ca^2+^ binding equilibrium dissociation constant (K_d, Ca2+_) extracted from a plot of maximal intensity versus CaCl_2_ concentration was 0.44 ± 0.4 mM, not significantly different from MCU-NTD_WT_ (*i.e.* K_d, Ca2+_ = 0.96 ± 0.7 mM), determined in the same manner (Figs. 7A, 7B, 7C and Table 4).

**Table 4:** Ca^2+^ binding of MCUb-NTD_WT_ and MCU-NTD_WT_.

	**Equilibrium dissociation constants for Ca**^**2+**^**(K**_**d, Ca2+**_**)**^a^			
	**Titration 1**	**Titration 2**	**Titration 3**	**Average ± SEM**
**MCUb-NTD**_**WT**_	0.18 ± 0.31	0.81 ± 0.80	0.53 ± 0.60	0.44 ± 0.40
**MCU-NTD**_**WT**_	2.92 ± 3.57	0.65 ± 0.59	0.56 ± 0.23	0.96 ± 0.68

a Data were fit using a one site binding model that takes into account protein concentration. Units are in mM.

We next used ANS binding to evaluate solvent-exposed hydrophobicity of the MCUb-NTD with and without Ca^2+^. As we previously reported [23], the addition of 5 mM Ca^2+^ to MCUb-NTD_WT_ increased ANS binding as indicated by the enhanced ANS fluorescence intensity and blue-shifted emission maximum (Fig. 7D) [61]. We next expressed and purified MCUb-NTD_D99A/E103A_ designed to disrupt Ca^2+^ coordination at the central helix. Notably, MCUb-NTD_D99A/E103A_ showed significantly increased ANS binding compared to the MCUb-NTD_WT_, indicating the central helix double mutation alone increases solvent-exposed hydrophobicity. Further, the addition of 5 mM Ca^2+^ to the MCUb-NTD_D99A/E103A_ sample did not cause any changes in structure, consistent with abrogation of Ca^2+^ binding. We also prepared a MCUb-NTD_M119R_ protein sample, observing remarkably analogous effects with and without Ca^2+^ as the MCUb-NTD_D99A/E103A_ protein, despite the mutation site on the opposite face of the protein compared to D99A/E103A (Figs. 7D and 7E).

We previously found Ca^2+^ does not change the secondary structure levels of MCUb-NTD_WT_ using far-UV circular dichroism (CD) spectroscopy [23] and similarly, did not observe any Ca^2+^-dependent changes here at 20 °C (Fig. 7F). Nevertheless, congruent with the mutation-dependent increases in solvent-exposed hydrophobicity, the D99A/E103A and M119R mutations significantly increased the negative ellipticity at 208 nm for both mutants and at 222 nm for the D99A/E103A mutant compared to WT, with neither mutation exhibiting Ca^2+^-dependent structural changes (Fig. 7F-7H).

Collectively, our *in vitro* light spectroscopic analyses using purified recombinant MCUb-NTD are remarkably congruent with the *in silico* data, showing abrogated Ca^2+^-dependent changes in solvent-exposed hydrophobicity upon direct mutation to central helix coordinating residues (*i.e.* D99A/E103A) or to co-evolving residues far from the central helix (*i.e.* M119R). Moreover, CD spectra show no changes in secondary structure due to Ca^2+^ binding either by WT or mutant MCUb proteins.

### 3.9 Solution NMR confirms D99, E103 and M119 belong to the same structural network of interacting residues

**Fig. 8:**
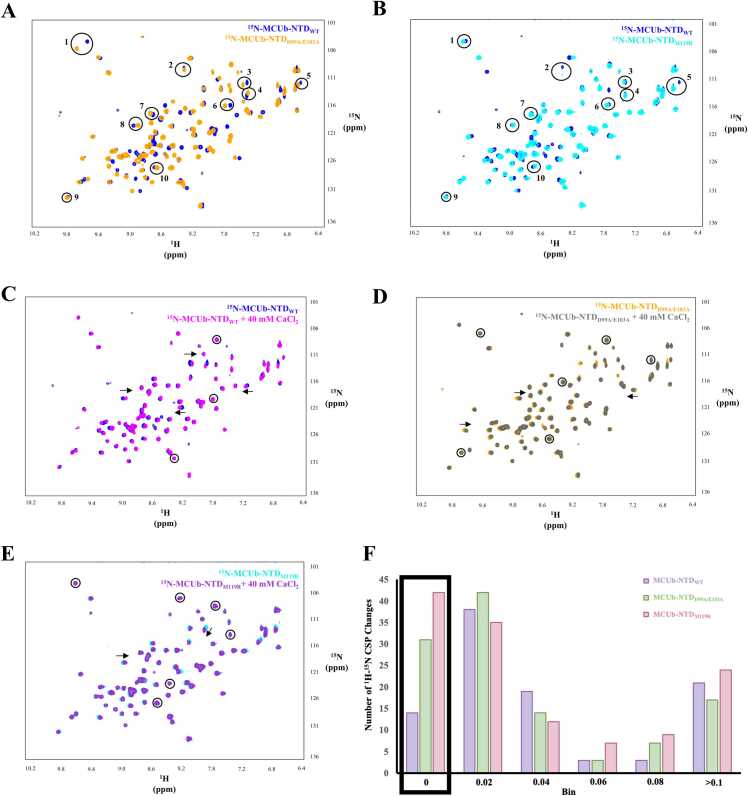
Structural assessment of MCUb-NTD_WT_ compared to MCUb-NTD_D99A/E103A_ and MCUb-NTD_M119R_ in the absence and presence of CaCl_2_. (A) ^1^H-^15^N HSQC spectra of ^15^N-MCUb-NTD_WT_ (blue) overlaid with ^15^N-MCUb-NTD_D99A/E103_ (orange) in the absence of CaCl_2_. (B) ^1^H-^15^N HSQC spectra of ^15^N-MCUb-NTD_WT_ (blue) overlaid with ^15^N-MCUb-NTD_M119R_ (cyan) in the absence of CaCl_2_. In (*A,B*) ten residue-specific chemical shift changes from the wild-type to mutant spectra are highlighted with a black circle and numbered. (C) ^1^H-^15^N HSQC spectra of ^15^N-MCUb-NTD_WT_ (blue) before and (magenta) after 40 mM CaCl_2_ supplementation. (D) ^1^H-^15^N HSQC spectra of ^15^N-MCUb-NTD_D99A/E103A_ (orange) before and (gray) after 40 mM CaCl_2_ supplementation. (E) ^1^H-^15^N HSQC spectra of ^15^N-MCUb-NTD_M119R_ (cyan) before and (violet) after 40 mM CaCl_2_ supplementation. (F) The number of ^1^H-^15^N CSP changes before and after 40 mM CaCl_2_ supplementation for each MCUb-NTD protein was plotted using bin widths of 0.02, with the last bin including all CSP changes greater than 0.1. The number of ^1^H(^15^N) crosspeaks that did not undergo CSPs after 40 mM CaCl_2_ supplementation were identified in the first bin, highlighted by a black box. In (*C-E*), residues undergoing large CSPs are highlighted with a black arrow, and residues not undergoing CSPs are highlighted with a black circle. All solution NMR spectroscopy experiments were performed using a 600 MHz Bruker 600 spectrometer at 20 ˚C.

We next assessed the structural consequences of Ca^2+^ binding to the MCUb-NTD_D99A/E103A_ and MCUb-NTD_M119R_ mutants compared to MCUb-NTD_WT_ by solution NMR spectroscopy. All ^1^H-^15^N-HSQC spectra of uniformly ^15^N-labeled MCUb-NTD_WT_ or mutant recombinant proteins in the absence or presence of 40 mM CaCl_2_ showed well-dispersed amide [^1^H(^15^N)] crosspeaks in the ∼6.5–10 ^1^H(^15^N) ppm range, indicating well-folded structures (Fig. 8A-8E). Spectra were acquired on each protein before and after supplementation with 40 mM CaCl_2_ to saturate all possible binding sites. Remarkably, the mutant spectra showed several of the same residue-specific chemical shift changes, consistent with D99/E103 and M119 being part of the same network of co-evolving residues (Figs. 8A and 8B). Chemical shift perturbations (CSPs) were apparent for each of the WT and mutant proteins after addition of 40 mM CaCl_2_, indicating divalent cation interactions and consistent with the MD simulations (Fig. 8C-8E). Since the ^1^H(^15^N) amide chemical shifts remain unassigned for MCUb-NTD, we tabulated the number of CSPs upon Ca^2+^ addition for each of the proteins as a measure of the mutation-specific changes in binding. Recall that in our MD simulations both D99A/E103A and M119R mutations abrogated Ca^2+^ interactions with the central helix, shifting the interactions instead to the conserved MRAP residues of D116 and D133, respectively, although with ∼2-fold less contacts (Table 3). Our solution NMR data showed ∼2-fold and ∼3-fold fewer unperturbed residues for ^15^N-MCUb-NTD_D99A/E103A_ and ^15^N-MCUb-NTD_M119R_, respectively, compared to ^15^N-MCUb-NTD_WT_, as reported by the number of ^1^H(^15^N) crosspeaks that did not change after CaCl_2_ addition and consistent with the disruption of the primary central helix Ca^2+^ binding site on MCUb-NTD (Fig. 8**F**).

Together, our NMR data confirm that central helix D99 and E103 are part of the same Ca^2+^-sensitive structural network of residues as M119 on the opposite face of MCUb-NTD. Further, the decreases in MCUb-NTD:Ca^2+^ binding upon D99A/E103A or M119R mutation are consistent with our *in silico* predictions suggesting the central helix is the primary Ca^2+^ interaction site for MCUb-NTD.

### 3.10 MCUb-NTD deletion or perturbation of the sector of co-evolving residues enhances mitochondrial Ca^2+^ uptake

**Fig. 9:**
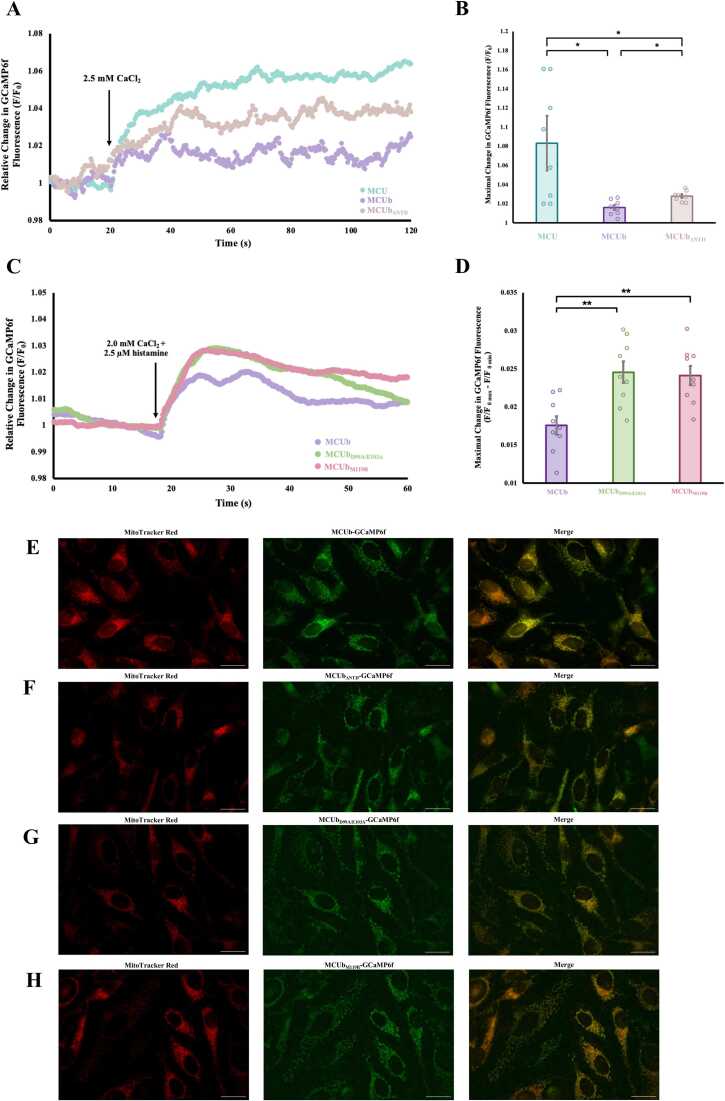
Mitochondrial Ca^2+^ uptake measured by GCaMP6f fluorescence. (A) Representative traces showing relative changes in mitochondrial matrix Ca^2+^ assessed by GCaMP6f fluorescence in permeabilized HEK293T cells overexpressing fused pEGFP-MCU-GCaMP6f, -MCUb-GCaMP6f, or -MCUb_ΔNTD_-GCaMP6f. The arrow indicates the addition of 2.5 mM CaCl_2_. (B) Welsch’s ANOVA followed by Games-Howell’s post-hoc test was used to compare the maximal change in GCaMP6f fluorescence, where *P < 0.05. In (*A, B*) data were acquired using a PTI QuantMaster spectrofluorometer (Horiba) equipped with electronic temperature control set to 22.5 °C. (C) Representative traces showing relative changes in mitochondrial matrix Ca^2+^ assessed by GCaMP6f fluorescence in intact HeLa cells overexpressing pEGFP-MCUb-GCaMP6f, -MCUb_D99A/E103A_-GCaMP6f, or -MCUb_M119R_-GCaMP6f fusion constructs. The arrow indicates the perfusion of 3 mL of Ca^2+^-free HEPES buffered saline solution (HBSS) supplemented with 2 mM CaCl_2_ and 2.5 µM histamine. (D) A one-way ANOVA and Tukey’s HSD post-hoc test were used to compare the maximal change in GCaMP6f fluorescence, where **P < 0.01. In (*C, D*), data were acquired using a Nikon Diaphot microscope equipped with a 40x objective (Nikon Fluor 40/1.30 Oil Ph4) used to locate ∼2–5 cells for visualization, with a maximum of three histamine responses per dish on distinct areas assessed per day over a maximum of two days. Epifluorescence microscopy images of live HeLa cells overexpressing (E) MCUb-GCaMP6f, (F) MCUb_ΔNTD_-GCaMP6f, (G) MCUb_D99A/E103A_-GCaMP6f or (H) MCUb_M119R_-GCaMP6f fusion constructs. In (*E-H*), MitoTracker Red (red) and GCaMP6f (green) images were acquired through a 63 × objective (Zeiss 63/1.4 Oil Plan Apochromat) after incubating the cells with 250 nM MitoTracker Red and bathing the cells in HBSS buffer supplemented with 5 mM CaCl_2_; scale bars are 30 μm. In (*A, B*), data are means ± SEM from n = 8 separate experiments and 8 transfections. In (*C, D*), data are means ± SEM from n = 9 separate experiments and 5 transfections.

Finally, to examine the functional role of the MCUb-NTD *in cellulo*, we monitored changes in mitochondrial matrix Ca^2+^ in permeabilized HEK293T cells overexpressing MCU-GCaMP6f, MCUb-GCaMP6f, or MCUb-GCaMP6f with a deleted NTD (*i.e.* MCUb_ΔNTD_-GCaMP6f) upon addition of 2.5 mM CaCl_2_ to the bathing medium. After the bolus of CaCl_2_, digitonin-permeabilized HEK293T cells overexpressing MCUb-GCaMP6f showed a significantly decreased maximal change in GCaMP6f fluorescence compared to cells overexpressing MCU-GCaMP6f (Figs. 9A and 9B). HEK293T cells transfected with MCUb_ΔNTD_-GCaMP6f showed a higher maximal change in GCaMP6f fluorescence compared to MCUb-GCaMP6f but significantly decreased compared to MCU-GCaMP6f (Figs. 9A and 9B).

While we note the possibility that expression differences may have contributed to the differences between MCUb_ΔNTD_-GCaMP6f and MCUb-GCaMP6f in the bulk permeabilized cell experiments, we believe this is unlikely since epifluorescence imaging shows MCUb_ΔNTD_-GCaMP6f and MCUb-GCaMP6f have similar transfection efficiencies as well as apparent expression levels and co-localization with MitoTracker Red in live HeLa cells (Figs. 9E and 9F). Further, the permeabilized cell data are fully consistent with our recent publication comparing histamine-induced mitochondrial Ca^2+^ uptake in intact HeLa cells expressing these constructs, where only cells specifically expressing these proteins were analyzed [23].

Given the demonstrated regulatory role of MCUb-NTD on mitochondrial Ca^2+^ uptake, we next generated point mutations to the central helix D99/E103 Ca^2+^-binding residues and the distinct M119 site on the opposite face of the protein but within the sector of co-evolving residues. We assessed mitochondrial Ca^2+^ uptake in intact HeLa cells overexpressing fused MCUb-GCaMP6f, MCUb_D99A/E103A_-GCaMP6f or MCUb_M119R_-GCaMP6f. Excitingly, the HeLa cells transfected with either MCUb_D99A/E103A_-GCaMP6f or MCUb_M119R_-GCaMP6f showed significantly increased histamine-induced maximal change in GCaMP6f fluorescence compared to MCUb-GCaMP6f (Figs. 9C and 9D). The mutations did not alter the MitoTracker Red-like localization, transfection efficiency or apparent expression levels of the MCUb-GCaMP6f fusion as assessed by live HeLa cell epifluorescence imaging (Figs. 9G and 9H).

Thus, our *in cellulo* data confirms that MCUb-NTD functions as a regulatory domain, where deletion suppresses the inhibitory efficacy of full-length MCUb; moreover, our functional data also indicates that Ca^2+^ binding to the MCUb-NTD central helix within the sector of co-evolving residues reinforces the inhibitory action of MCUb-NTD as direct disruption via D99A/E103A mutation or indirect perturbation of the sector via M119R relaxes the inhibitory effects.

## 4 Discussion

We previously identified the MCU regulating acidic patch (MRAP) as a contiguous, electronegatively charged surface region on the MCU-NTD, primarily made up of D131, D147 and D148, that interacts with Mg^2+^ and Ca^2+^. Upon binding of either divalent cation, a disruption of domain self-association occurs. HeLa cells over-expressing MCU with either D131A/R or D147A/R mutants showed suppressed maximal mitochondrial Ca^2+^ uptake concomitant with decreased higher order oligomerization; moreover, supraphysiological loading of the mitochondrial matrix with Ca^2+^ or Mg^2+^ inhibited maximal Ca^2+^ uptake [29]. Interestingly, divalent cation binding to MRAP also mediates changes in the Ca^2+^ sensing and regulatory properties of MICU1, implying an allosteric coupling from the MCU-NTD through the transmembrane domains (TMDs) to the IMS side of the channel harbouring MICU1 [28]. The known post-translational modifications on the MCU-NTD further reinforce the domain as a hub for receiving regulatory inputs. For example, we contributed to work showing *S*-glutathionylation of the MCU-NTD C97 promotes domain as well as channel oligomerization, increasing MCU-mediated mitochondrial Ca^2+^ uptake [62]. Additionally, Pyk2-dependent Y158 and AMPK-dependent S57 phosphorylation of the MCU-NTD increases mitochondrial Ca^2+^ uptake, while CAMKII-mediated S92 phosphorylation of the MCU-NTD disrupts dimerization of tetrameric channels [27], [63], [64]. Most recently, we demonstrated that the MCU-NTD tightly interacts with the MCUb-NTD with sub-μM affinity, promoting (*i*) integration of MCUb into mtCU and (*ii*) inhibitory efficacy of MCUb [23].

While MCU-NTD has been established as a regulatory hub receiving multiple disparate inputs, the molecular mechanisms underlying mtCU modulation via the highly homologous MCUb-NTD remain enigmatic. Here, we used *in silico*, *in vitro* and *in cellulo* experiments to study how divalent cations interact with the MCUb-NTD and the associated effects on the biophysical and structural properties as well as inhibitory function of the domain. The human MCUb-NTD structure has proven refractory to high-resolution structural determination. Nevertheless, given the high sequence similarity and identity to MCU-NTD, MCUb-NTD is an excellent candidate for homology modeling. Thus, we determined a 1.6 Å MCU-NTD X-ray crystal structure in the absence of divalent cations and used this atomic-resolution structure to homology model MCUb-NTD; moreover, both structures were used in 1 *μs* all atom MD simulations with and without Ca^2+^ or Mg^2+^.

Our simulations revealed disparate residues involved in divalent cation binding for the two paralogs. Whereas Ca^2+^ and Mg^2+^ preferentially interacted *in silico* with the MRAP residues on MCU-NTD, these cations were primarily attracted to negatively charged residues of the MCUb-NTD β-grasp fold central helix (*i.e.* D99 and E103). This difference is consistent with non-conserved acidic interacting residues at the central helix and MRAP residues for MCU-NTD and MCUb-NTD, respectively (*i.e.* D131/D147/D148 in MCU MRAP exist as D116/N132/D133 in MCUb and D99/E103 in the MCUb central helix exist as Q114/E118 in MCU). It is noteworthy that for MCU-NTD, Ca^2+^ and Mg^2+^ showed specific interactions with MRAP when placed randomly in the simulation box, consistent with past *in vitro* and *in cellulo* work identifying and characterizing the interactions in MRAP [28], [29]. Further, pySCA reinforced these differences, highlighting that central helix D99/E103 (MCUb) and Q114/E118 (MCU) of both proteins co-evolved within a larger network of residues that includes M119 in MCUb and the aligned R134 in MCU. Indeed, despite M119 (MCUb) and R134 (MCU) localization on the opposite face of the domain compared to the central helix, the M119R mutation moved divalent cation binding from the central helix to the MCUb MRAP residues in contrast to the R134M mutation in MCU where the preference for Ca^2+^ and Mg^2+^ binding in MRAP remained unaltered compared to WT.

While the *in silico* divalent cation binding observed at the MCU-NTD MRAP was congruent with past *in vitro* work, we used purified recombinant MCUb-NTD to similarly validate the Ca^2+^ interactions. We found that the apparent Ca^2+^ binding affinities for MCU-NTD and MCUb-NTD were ∼0.96 and 0.44 mM, respectively, relevant to the ∼mM local concentrations of Ca^2+^ that would occur near the exiting regions of Ca^2+^ channel pores [65], [66], [67]. Indeed, supplementation with Ca^2+^ altered the tertiary structure of MCUb-NTD, increasing ANS binding. Importantly, both the MCUb-NTD D99A/E103A central helix and M119R pySCA sector mutations, similarly abrogated the Ca^2+^ sensitivity in the ANS experiments while both promoted increased levels of α-helicity. Notably, this increased secondary structure is in-line with the enhanced central helix ideality for D99A/E103A and M119R mutants compared to MCUb-NTD_WT_, where the D99A/E103A mutant showed greater α-helicity enhancement in the CD and greater increase in ideality in the MD. Our solution NMR experiments confirmed the alterations in structure caused by the mutations and the Ca^2+^ interactions with the domain. More importantly, the D99A/E103A and M119R mutants indeed showed fewer but not completely abrogated CSPs caused by Ca^2+^, consistent with a perturbation of binding at the central helix and shifting of interactions to other sites on the protein as revealed by the MD.

Functionally, the role that Ca^2+^ binding to the MCUb-NTD may play in regulating the inhibitory efficacy was also examined *in cellulo*. Expression of MCU with a deleted NTD suppresses mtCU activity [27]. Conversely, we showed that deletion of the NTD in MCUb reduces co-localization of MCUb with MCU and enhances agonist-induced mtCU function in HeLa cells [23]. Here, we similarly assessed the role of the MCUb-NTD in mtCU activity using permeabilized HEK293T cells without the need for upstream agonist stimulation. Indeed, a simple bolus of Ca^2+^ addition to the bathing medium confirmed that cells overexpressing MCUb with a deleted NTD uptake Ca^2+^ to higher levels compared to cells overexpressing WT MCUb. Further, MCUb harboring either the D99A/E103A or the M119R mutations similarly showed enhanced Ca^2+^ uptake. Higher levels of mitochondrial Ca^2+^ uptake were also observed in intact cell experiments, where MCUb D99A/E103A or MCUb M119R overexpressing HeLa cells showed higher histamine-induced peak mitochondrial Ca^2+^ levels compared to WT MCUb overexpressing cells.

Interestingly, MCU-NTD_WT_ showed decreased structural change and internal motion, measured by RMSD and S^2^, respectively, at 320 K (*i.e*. ∼47 ˚C) compared to 310 K (*i.e*. ∼37 ˚C) in contrast to MCUb-NTD_WT_, exhibiting the opposite temperature-dependent effects *in silico*. The contrasting temperature effects on MCU-NTD and MCUb-NTD are coherent with energetic and thermogenic considerations of the cell. Namely, mitochondrial Ca^2+^ uptake enhances dehydrogenase activity of the TCA cycle and thus, aerobic metabolism by promoting electron transfer, proton gradient across the IMM and ATP production. Further, MCU can form a unique complex that replaces MICU1 with uncoupling protein-1 (UCP1), accelerating mitochondrial Ca^2+^ uptake but also promoting uncoupled respiration by UCP1 for adaptive thermogenesis [68]. In contrast, diminished respiratory chain activity is associated with increased MCUb expression and diminished mtCU activity [24]. Thus, the enhanced MCU-NTD but reduced MCUb-NTD stability at high temperature may be an adaption to meet the ATP and/or heat production demands of the cell.

## 5 Conclusions

**Fig. 10:**
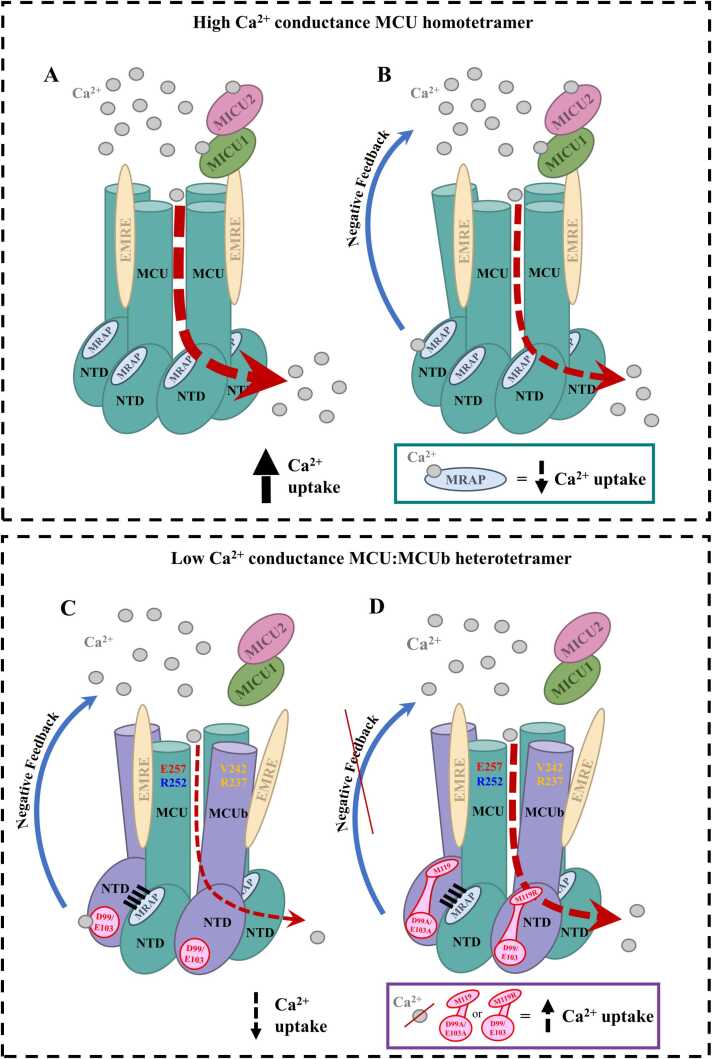
Models of divalent cation autoregulatory mechanisms in MCU- and MCUb-NTD mediated decreases in mtCU Ca^2+^ conductance. (A) A single homotetrameric MCU channel of mtCU shows high Ca^2+^ uptake under high IMS Ca^2+^ conditions. (B) Ca^2+^ binding to MRAP residues of the MCU-NTD destabilizes the channel, decreasing Ca^2+^ conductance [29], [74]. (C) A single heterotetrameric MCU:MCUb channel with tight MCU-NTD:MCUb-NTD interactions, perturbing MRAP of MCU (black bars) [23]. Ca^2+^ binding to the central helix residues of MCUb-NTD (*i.e.* D99 and E103) is proposed to contribute to the inhibitory function of MCUb, causing negative feedback (blue arrow) and decreased Ca^2+^ conductance. (D) The inhibitory action of Ca^2+^ binding to D99 and E103 on the central helix of MCUb-NTD is abrogated by direct mutation to the distinct Ca^2+^ selective coordinating residues or mutation to a distinct site on the opposite face of MCUb-NTD (*i.e.* M119R) also within the functional sector of co-evolving residues identified by pySCA [31], [46]. In (*C-D*), two critical residue substitutions identified within the TM regions of MCUb (*i.e.* R252 and E257 within MCU corresponding to W237 and V242 in MCUb) [25], [26], [72] and changes in the pore architecture and assembly with regulatory components [26], [70], [75] are two mechanisms contributing to the low MCUb-mediated Ca^2+^ conductance. In (*A-D*), the divalent cation binding site, MRAP (blue), is shown on MCU (teal), and D99/E103 (pink) is shown on MCUb (purple) within the respective N-terminal domains (NTDs). EMRE (beige) is shown at less than a 1 ×EMRE:1 ×MCU/MCUb stoichiometry, with MICU1 (green) and MICU2 (pink) on the IMS side of the channel. The thick and thin red dashed lines depict high to low Ca^2+^ conductance, respectively, with Ca^2+^ ions shown as gray spheres.

Our work reveals that Ca^2+^ binding to the MCUb-NTD supports the inhibitory action of MCUb. Homotetramerized MCU subunits form maximally active mtCU channels (Fig. 10A). We and others previously showed that Ca^2+^ or Mg^2+^ binding to MCU MRAP residues in the matrix inhibit mtCU channel activity (Fig. 10B) [28], [29], [69]. Here, we found that the MRAP residues are not among the large network of co-evolving MCU-NTD residues, and thus, mutation to this sector (*i.e.* R134M) did not affect interactions with Ca^2+^. Nevertheless, we recently showed MCU-NTD MRAP is a critical site for interactions with MCUb-NTD [23], contributing to integration of MCUb into mtCU and the inhibitory efficacy in cells expressing MCUb (Fig. 10C). While interactions of Ca^2+^ with MCU-NTD MRAP may be impeded due to MRAP-mediated hetero-associations with MCUb-NTD, our work here reveals a distinct Ca^2+^ binding site on the central helix of MCUb-NTD made up of residues D99 and E103 (Fig. 10C). These residues are part of the major network of co-evolving MCUb-NTD residues, and indeed, direct D99A/E103A or M119R, both perturbed Ca^2+^ coordination and enhanced mitochondrial Ca^2+^ uptake in our studies (Fig. 10D). Therefore, we propose that disparate residues involved in Ca^2+^ sensing by the MCU-NTD and MCUb-NTD contribute to inhibitory roles in mtCU function. It is tempting to speculate that divalent cation-dependent conformational changes induced by MCUb-NTD propagate through the TMDs, contributing to the altered interaction of MCUb with EMRE [26] and ultimately the absent interaction between MCUb and MICU1 [70]. Consistent with this notion, the TMDs of the MCU and MCUb paralogs have two critical residue differences identified near the DIME motif suggested to affect Ca^2+^ uptake propensities (*i.e.* R252 and E257 in MCU correspond to W237 and V242 in MCUb) (Figs. 10C and 10D) [25], [26], [71], [72].

Overall, our work here presents new insights into the mechanisms underlying MCUb-mediated low Ca^2+^ conductance of mtCU and provides a unique framework for future hypotheses and drug design approaches.

## CRediT authorship contribution statement

**Taylor Lake:** Methodology. **Megan Noble:** Methodology, Conceptualization. **Peter Stathopulos:** Writing – review & editing, Validation, Supervision, Resources, Project administration, Funding acquisition, Data curation, Conceptualization. **Murray Junop:** Writing – review & editing, Supervision, Resources, Conceptualization. **Ryan Grainger:** Writing – review & editing, Methodology, Formal analysis. **Danielle M. Colussi:** Writing – review & editing, Writing – original draft, Visualization, Validation, Methodology, Investigation, Formal analysis, Conceptualization.

## Declaration of Competing Interest

The authors declare that they have no known competing financial interests or personal relationships that could have appeared to influence the work reported in this paper.

## Data Availability

The 1.6 Å crystal structure coordinates are freely available for download from the Protein Data Bank under the accession code 8URG.pdb. The MD trajectories generated in this study are freely available from zenodo.org under the accession links 10.5281/zenodo.X, where X is 14237186, 14237025, 14236581, 14236378, 14235881, 14232618, 14232544, 14232451, 14232357, 14232153, 14231988, 14231741, 14231573, 14231396, 14231183, 14231124, 14230957, 14230246, 14229735, 14268024 or 14229869 to access the twenty-one different GROMACS 2022.3 trajectory (xtc) and energy (edr) files (1000 ns each). All other data available upon reasonable request to the corresponding author (pstatho@uwo.ca).

## Appendix A Supplementary material

Supplementary Video descriptions
