# Supplementary material for: Disrupting the network of co-evolving amino terminal domain residues relieves mitochondrial calcium uptake inhibition by MCUb
Source: Comput Struct Biotechnol J. 2024 Dec 12;27:190–213. doi: 10.1016/j.csbj.2024.12.007 (PMC11867204; doi:10.1016/j.csbj.2024.12.007)
Supplement: Supplementary file 22 — Supplementary Video descriptions [file mmc1.docx]

**Supplemental Videos**

The mp4 file movie outputs were generated in ChimeraX ^44^. All simulations were conducted at 310 K (~37 °C) unless otherwise indicated, for 1 µs duration. The MCU-NTD structure is shown in light teal and MCUb-NTD is shown in light purple. The Ca^2+^ ions are shown as gray spheres and Mg^2+^ ions are depicted as yellow spheres. All hydrogen atoms were removed from the MD videos.

**MCU-NTD_WT_ and MCUb-NTD_WT_ Temperature-Dependent MD Simulations**

**Video S1.** Dynamics of our newly resolved MCU-NTD_WT_, residues 74 to 165, in the solvated system at 310 K (~37 °C). The six β-sheets and two α-helical regions are highlighted in dark teal, with the connecting seven loop regions shown in beige. The loop regions show greater dynamics than the secondary structure, indicated by lower S^2^ order parameters within loops.

**Video S2.** Increased stability of MCU-NTD_WT_ in the solvated system at a higher temperature of 320 K (~47 °C). The six β-sheets and two α-helical regions are highlighted in light teal, with the connecting loop regions shown in beige. The higher temperature decreased the dynamics, measured by S^2^ order parameter, of the central helix and loop 5.

**Video S3.** Dynamics of homology modeled MCUb-NTD_WT_, residues 59 to 150, in the solvated system at 310 K (~37 °C). The six β-sheets and two α-helical regions are highlighted in dark purple, with the connecting loop regions shown in beige. The average RMSD of the 92 Cα atoms over the 1 μs simulation conducted at 310 K (~37 °C) was lower for MCUb-NTD_WT_ compared to MCU-NTD_WT_, indicating decreased conformational change over the simulation.

**Video S4.** Decreased stability of MCUb-NTD_WT_ in the solvated system at a higher temperature of 320 K (~47 °C), contrary to MCU-NTD_WT_. The six β-sheets and two α-helical regions are highlighted in light purple, with the connecting loop regions shown in beige.

**MCU-NTD_WT_ and MCUb-NTD_WT_ MD Simulations with Divalent Cations**

**Video S5.** With 1 Ca^2+^ ion added into the solvated system of MCU-NTD_WT_, the Ca^2+^ ion underwent ~97 % of total protein interactions with MRAP residues D131, D147 and D148 (*i.e.* 29,851, 29,594 and 32,478 interactions, respectively) over the 1 µs simulation. Residues D131, D147 and D148 are shown as sticks in red.

**Video S6.** MCU-NTD_WT_ with 5 Ca^2+^ ions added into the solvated system showed 1 Ca^2+^ ion interacting to MRAP residues D131, D147, and D148 similarly to the MCU-NTD_WT_ system with 1 Ca^2+^ ion, and three additional Ca^2+^ ions interacting predominantly with E118, D123 and D155. The interaction of Ca^2+^ with E118 destabilized the central helix. Residues E118, D123, D131, D147, D148 and D155 are shown as sticks in red.

**Video S7.** With 1 Ca^2+^ ion added into the solvated system of MCU-NTD_WT_ at 320 K (~47 °C), the Ca^2+^ ion underwent ~94 % of total protein interactions with MRAP residues. However, ~3-fold fewer interactions were observed with D131 at 320 K (~47 °C) compared to 310 K (~37 °C) over the 1 µs simulation. Residues D131, D147 and D148 are shown as sticks in red.

**Video S8.** With 1 Mg^2+^ added into the MCU-NTD_WT_ solvated system there were ~24-fold fewer interactions with the protein, with ~39 % identified with D131 and D147 of MRAP and ~28 % identified with E118 of the central helix, with Mg^2+^ hopping from the central helix to MRAP. Residues E118, D131, D147 and D148 are shown as sticks in red.

**Video S9.** Adding 5 Mg^2+^ ions into the MCU-NTD_WT_ solvated system increased the interactions of Mg^2+^ with MRAP D131 and D147 but did not affect interactions with E118. Additional Mg^2+^ ions increase the occupancy in MRAP. Residues E118, D131, D147, and D148 are shown as sticks in red.

**Video S10.** Homology modeled MCUb-NTD_WT_ shown with 1 Ca^2+^ ion added into the solvated system. Instead of interacting with MRAP residues, the Ca^2+^ ion interacted with D99 and E103 of the central helix, showing 29,890 and 30,653 interactions, respectively. Residues D99 and E103 are shown as sticks in red.

**Video S11.** MCUb-NTD_WT_ in the presence of 5 Ca^2+^ ions showed 1 Ca^2+^ ion similarly interacting with D99 and E103, while two of the additional Ca^2+^ ions predominantly interacted with D116 and D133 of MRAP in MCUb. Residues D99, E103, D116 and D133 are shown as sticks in red.

**Video S12.** With 1 Mg^2+^ ion was added into the MCUb-NTD_WT_ solvated system there were ~10-fold fewer interactions with the protein. The Mg^2+^ ion predominantly interacted with D99 and E103 of the central helix, with a small portion of interactions occurring with D116 and D133. Residues D99, E103, D116 and D133 are shown as sticks in red.

**Video S13.** Similar to 1 Mg^2+^, when 5 Mg^2+^ ions were added into the MCUb-NTD_WT_ solvated system, predominant interactions occurred with D99 and E103 and fewer interactions occurred with D116 and D133. Residues D99, E103, D116 and D133 are shown as sticks in red.

**MD Simulations of MCU- and MCUb-NTD with Mutation to Distinct Preferential Divalent Cation Coordinating Sites**

**Video S14.** With MRAP residues in MCU-NTD mutated to Ala (*i.e.* D131A, D147A, and D148A), shown as sticks in orange, a single Ca^2+^ ion added into the solvated system did not interact with the MRAP region but rather showed 29,676 interactions with D155 in loop 7 (L7). Residue D155 is shown with sticks in red.

**Video S15.** The conserved MRAP residues in MCUb-NTD akin to MCU were mutated to Ala (*i.e.* D116A and D133A in MCUb correspond to D131 and D148 in MCU, respectively), shown as sticks in orange. The D116A and D133A double mutation in MCUb-NTD did not disrupt the preferential interaction between the Ca^2+^ ion and D99 and E103, shown as sticks in red, on the central helix of the MCUb-NTD_D116A/D133A_ protein.

**Video S16.** Dynamics of MCUb-NTD_D99A/E103A_ over the simulation indicate the central helix became less dynamic along with MRAP residues, measured by S^2^. Mutated residues D99A/E103A are shown as sticks in orange, and residues D116/D131 are shown as sticks in red.

**Video S17.** The MCUb-NTD_D99A/E103A_ double mutation resulted in a swap of the coordination site of Ca^2+^ from the central helix to D116, one of the conserved MRAP residues (*i.e.* D131 in MCU). Mutated residues D99A/E103A are shown as sticks in orange, and residues D116/D131 are shown as sticks in red.

**pySCA-Driven MCU-NTD_R134M_ and MCUb-NTD_M119R_ MD Simulations**

**Video S18.** MCU-NTD_R134M_ showed reduced total backbone and central helix RMSDs compared to MCU-NTD_WT_. The dynamics of R134M decreased, indicated by increased S^2^. The mutated R134M residue is shown as sticks in pink, with MRAP residues D131, D147 and D148 shown as sticks in red.

**Video S19.** The 1 Ca^2+^ ion primarily interacted with MRAP residues D147 and D148 but exhibited minimal interactions with D131. The mutated R134M residue is shown as sticks in pink, with MRAP residues D131, D147 and D148 shown as sticks in red.

**Video S20.** The dynamics of MCUb-NTD_M119R_ in the solvated system highlighted the central helix RMSD increasing relative to ideal geometry, consistent with residues of the central helix being part of the same sector as M119R identified by pySCA. The mutated M119R residue is shown as sticks in pink, with residues D99 and E103 on the central helix shown as sticks in red, along with the corresponding MRAP residues D116 and D133.

**Video S21.** With 1 Ca^2+^ ion added into the solvated system with MCUb-NTD_M119R_, the Ca^2+^ ion predominantly interacted with D133 instead of D99 and E103. The mutated M119R residue is shown as sticks in pink, with residues D99 and E103 on the central helix shown as sticks in red, along with the corresponding MRAP residues D116 and D133.
