# Supplementary material for: Disrupting the network of co-evolving amino terminal domain residues relieves mitochondrial calcium uptake inhibition by MCUb
Source: Comput Struct Biotechnol J. 2024 Dec 12;27:190–213. doi: 10.1016/j.csbj.2024.12.007 (PMC11867204; doi:10.1016/j.csbj.2024.12.007)
Supplement: Supplementary file 23 — Supplementary Table S1, Figure S1 and Figure S2 [file mmc2.pdf]

Table S1. Residues constituting the single functional sectors of the MCU- and MCUb-NTDs defined using pySCA.

Protein	Sector	IC[a]	Residue number
**MCU-NTD**	**1**	**1**	81+85+104+114+135+139+142
		**2**	82+84+90+91+99+103+108+119+149+154+160
		**3**	92+93+95+97+118+121+122+144+163
		**4**	88+89+101+110+134+136+141+155
		**5**	76+83+86+102+111
**MCUb-NTD**	**1**	**1**	66+70+99+120+124+127
		**2**	67+69+76+82+84+88+93+104+134+143+145
		**3**	77+78+80+103+106+107+129
		**4**	73+74+86+95+119+121+126+140
		**5**	61+68+71+85+87+96

[a] Independent component (IC)

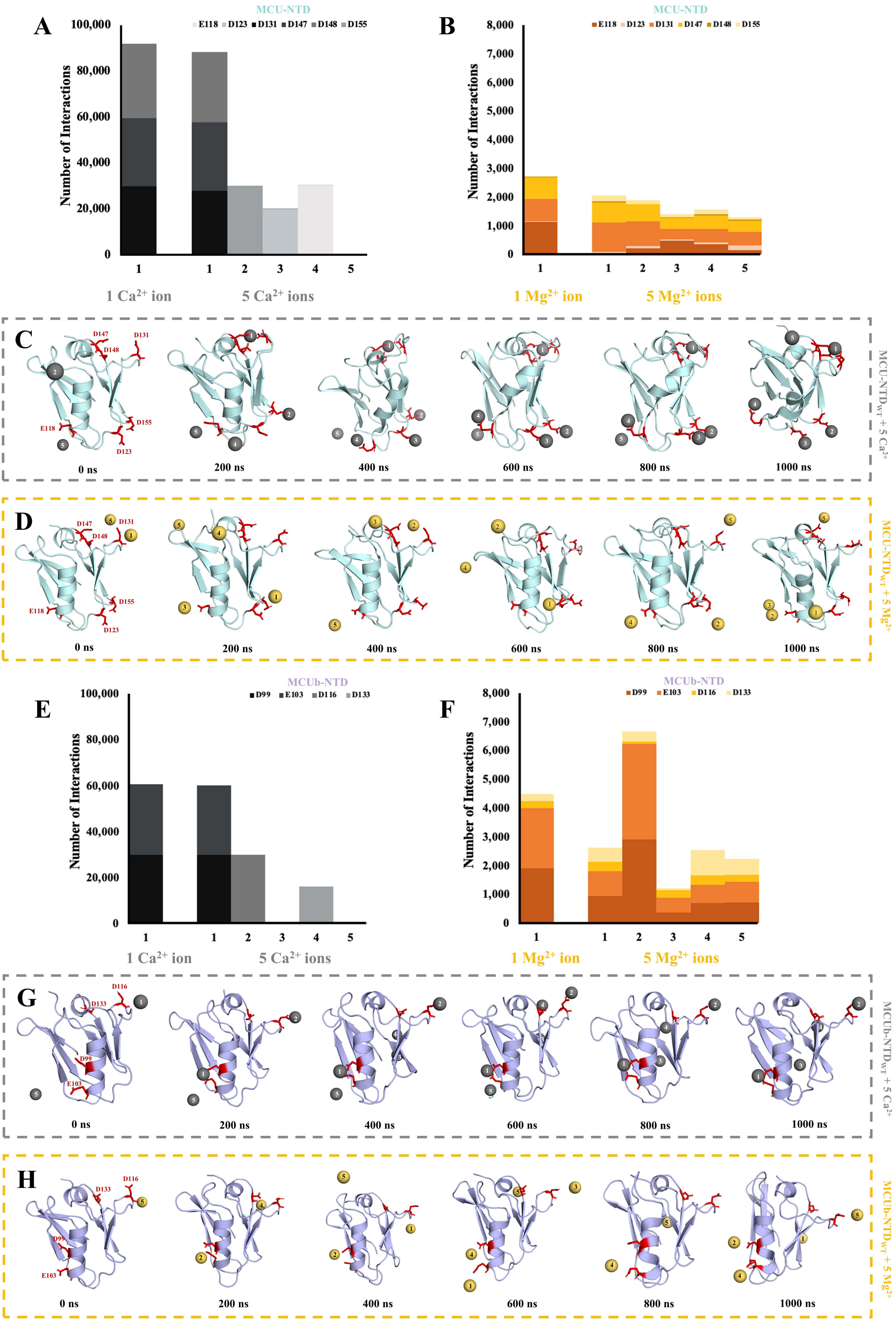

A
MCU-NTD
E118 D123 D131 D147 D148 D155
Number of Interactions
100,000
80,000
60,000
40,000
20,000
0
1 1 2 3 4 5
1 Ca2+ ion
5 Ca2+ ions
B
MCU-NTD
E118 D123 D131 D147 D148 D155
8,000
7,000
6,000
5,000
4,000
3,000
2,000
1,000
0
1 Mg2+ ion
5 Mg2+ ions
C
MCU-NTDWT + 5 Ca2+
0 ns
200 ns
400 ns
600 ns
800 ns
1000 ns
D
MCU-NTDWT + 5 Mg2+
E
MCUb-NTD
D99 E103 D116 D133
F
MCUb-NTD
G
MCUb-NTDWT + 5 Ca2+
H
MCUb-NTDWT + 5 Mg2+

Figure S1. Divalent cation interactions with MCU-NTD and MCUb-NTD. The number of interactions between the **(A)** 1 or 5 Ca^{2+} ions and **(B)** 1 or 5 Mg^{2+} ions added into the solvated MCU-NTD_{WT} system and residues E118, D123, D131, D147, D148, and D155 of MCU-NTD are indicated over the 1 μs simulations. Snapshots were taken every 200 ns from the 1 μs simulation with **(C)** 5 Ca^{2+} ions or **(D)** 5 Mg^{2+} ions added into the MCU-NTD_{WT} (teal) system. The electronegative residues E118, D123, D131, D147, D148, and D155 of MCU are shown as sticks in red. The number of interactions between the **(E)** 1 or 5 Ca^{2+} ions and **(F)** 1 or 5 Mg^{2+} ions and D99, E103, D116, and D133 of MCUb-NTD are also indicated over the 1 μs simulations. Snapshots were taken every 200 ns from the 1 μs simulation with **(G)** 5 Ca^{2+} ions or **(H)** 5 Mg^{2+} ions added into the MCUb-NTD_{WT} (purple) system. The electronegative residues D99, E103, D116, and D133 of MCUb are shown as sticks in red. In (*C, G*), the Ca^{2+} ions are shown in gray and numbered according to (*A, E*), respectively. In (*D, H*), the Mg^{2+} ions are shown in yellow, with each ion numbered according to (*B, F*), respectively. In (*A, B, E, F*), the number of interactions was taken as the sum of the ≤ 4 Å distances over 1 μs in 100 ps increments between any atom on the residue of interest and the divalent cation.

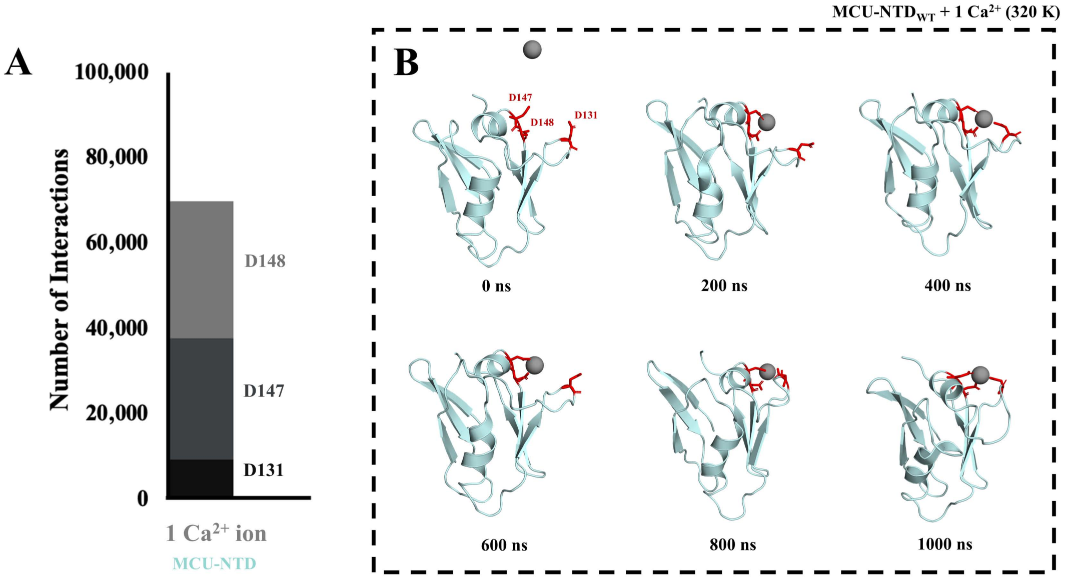

A
100,000
80,000
60,000
40,000
20,000
0
Number of Interactions
D148
D147
D131
1 Ca^{2+} ion
MCU-NTD
MCU-NTD$_{WT}$ + 1 Ca^{2+} (320 K)
B
D147
D148
D131
0 ns
200 ns
400 ns
600 ns
800 ns
1000 ns

Figure S2. Ca^{2+} interactions with MCU-NTD at 320 K. (A) The number of interactions between the 1 Ca^{2+} ion added into the solvated MCU-NTD_{WT} system and D131, D147 over D148 over the 1 μs simulation. **(B)** Snapshot images at 200 ns increments, highlighting the relative locations and contacts of D131, D147 and D148 MRAP side chains (red sticks) and the single Ca^{2+} ion (grey sphere) in the system. In (*A*), the number of interactions was taken as the sum of the ≤ 4 Å distances over 1 μs in 100 ps increments between any atom on the residue of interest and the divalent cation.
